# Improving the reliability of small- and wide-angle X-ray scattering measurements of anisotropic precipitates in metallic alloys using sample rotation

**DOI:** 10.1107/S1600576724009294

**Published:** 2024-11-04

**Authors:** Thomas Perrin, Gilbert A. Chahine, Stéphan Arnaud, Arthur Després, Pierre Heugue, Alexis Deschamps, Frédéric De Geuser

**Affiliations:** a SIMaP Université Grenoble Alpes, CNRS, Grenoble INP 38000Grenoble France; b Institut Néel 25 Avenue des Martyrs 38042Grenoble France; c DT/MPE, Safran Transmission Systems, 18 Boulevard Louis Seguin, 92707Colombes, France; Argonne National Laboratory, USA

**Keywords:** small- and wide-angle X-ray scattering, SAXS/WAXS, anisotropy, methodology, rotation, metallic alloys

## Abstract

Nanometric precipitates in metallic alloys often have highly anisotropic shapes. Given the large grain size and non-random texture typical of these alloys, performing small- and wide-angle X-ray scattering (SAXS/WAXS) measurements on such samples for determining their characteristics (typically size and volume fraction) results in highly anisotropic and irreproducible data. Rotations of flat samples during SAXS/WAXS acquisitions are presented here as a solution to these anisotropy issues. Two aluminium alloys containing anisotropic precipitates are used as examples to validate the approach with a −45°/45° angular range. Clear improvements can be seen on the SAXS *I*(*q*) fitting and the consistency between the different SAXS/WAXS measurements. This method­ology results in more reliable measurements of the precipitate’s characteristics, and thus allows for time- and space-resolved measurements with higher accuracy.

## Abstract 2

Rotations of small- and wide-angle X-ray scattering samples during acquisition are shown to give a drastic improvement in the reliability of the characterization of anisotropic precipitates in metallic alloys.

## 1. Introduction

Adding nanoscale precipitates to metallic alloys is one of the most efficient ways to improve their strength. The level of strengthening is intimately related to the size, morphology and volume fraction of the precipitates formed. These parameters are themselves controlled by the kinetic pathway used for the precipitation reaction (alloy composition, heat treatments) (Deschamps & Hutchinson, 2021 ▸). Therefore, great efforts have been made to quantify precipitation states at the nanometre scale in structural alloys (aluminium, steels, nickel-based *etc*.). For this purpose, a variety of experimental tools are available.

Transmission electron microscopy (TEM) observations are most frequently used for such analysis but are highly time consuming and give information on a very local scale (Chen *et al.*, 2009 ▸; Sharma *et al.*, 2009 ▸; Mousavi Anijdan *et al.*, 2021 ▸).

Atom probe tomography has also become a common tool for precipitate characterization and is particularly well suited to determining the chemistry of the nanophases (Biswas *et al.*, 2014 ▸; Ioannidou *et al.*, 2020 ▸) together with that of the surrounding matrix (Rementeria *et al.*, 2017 ▸; Wang *et al.*, 2024 ▸). However, its use for quantitative characterization of a precipitate’s state is limited by the very small probed volumes, and its use for precisely measuring nanoprecipitate sizes is strongly impeded by local magnification effects (Miller & Hetherington, 1991 ▸; Vurpillot *et al.*, 2001 ▸).

The characterization method that enabled the first observation of precursors for hardening precipitates in aluminium alloys is small-angle X-ray scattering (SAXS) (Guinier, 1952 ▸). This technique allows measurement of the volume fraction and size of the precipitates forming in a matrix by fitting with appropriate models the X-ray intensity scattered by the sample in transmission mode. The faster acquisition enabled by the use of synchrotron light sources and by the new generations of fast detectors has promoted time-resolved *in situ* studies (Deschamps *et al.*, 2012 ▸; Tsao *et al.*, 2013 ▸; Zhang *et al.*, 2016 ▸; Andrews *et al.*, 2017 ▸; Gumbmann *et al.*, 2017 ▸). In addition, the use of small beam sizes and short measurement times has enabled spatially resolved studies for mapping heterogeneous microstructures. For instance, various studies on friction stir welds in aluminium alloys were carried out using such SAXS mapping (Dumont *et al.*, 2006 ▸; Steuwer *et al.*, 2011 ▸; De Geuser *et al.*, 2014 ▸; dos Santos *et al.*, 2018 ▸).

If the SAXS signal is isotropic in reciprocal space, so will be the signal measured on the 2D detector. In this case, the radial mean computed to obtain the classical *I*(*q*) plot, with *I* being the intensity of the scattered beam and *q* the scattering vector [*q* = (4π/λ) sin(θ), where θ is half the scattering angle and λ is the wavelength of the incident radiation], is representative of the microstructure. Models can then be fitted over the *I*(*q*) plot, assuming the morphology of the particles. Isotropic signals are typically obtained for spherical particles (De Geuser & Deschamps, 2012 ▸; dos Santos *et al.*, 2018 ▸; Haas *et al.*, 2018 ▸), or particles with anisotropic morphology but with a random orientation distribution and a sufficient number density (*i.e.* a powder sample in the crystallographic sense) (Deschamps *et al.*, 2011 ▸). For the latter, different models can be used to fit the *I*(*q*) plots, such as the empirical model of Hammouda (2010 ▸).

However, particles with non-spherical morphology and non-random orientation give rise to an anisotropic signal whose radial integration does not provide a faithful representation of the microstructure. In the case of single crystals with precipitates having specific orientation relationships with the matrix, the contribution of each particle orientation can be treated separately to obtain relevant information (Fratzl *et al.*, 1993 ▸; Chang *et al.*, 2015 ▸); a relaxed case is when only limited misorientations exist in the illuminated volume between precipitates (Paris *et al.*, 1994 ▸; De Geuser *et al.*, 2012 ▸).

This leaves a substantial gap for alloys with anisotropic particles with ill-defined orientations with respect to the X-ray beam, or with orientations that are different but not different enough for the material to be considered as a powder. This is typically the case for samples of aluminium alloys with a large grain size comparable to the beam diameter and a non-random texture, in which precipitates with large aspect ratios can be found. Good examples of such situations are Al–Cu-bearing alloys (containing θ′, S or T_1_ precipitates in, respectively, Al–Cu, Al–Cu–Mg and Al–Cu–Li alloys). In such systems, obtaining a good sampling of precipitate orientations with respect to the incoming beam is a difficult task. Some averaging can be achieved by measuring many different sample locations, but it is often insufficient given the strong crystallographic texture of these materials, generally a consequence of rolling.

Simultaneously to SAXS measurements, wide-angle X-ray scattering (WAXS) can be measured to give information on the nature of the phases present in the matrix (Zhang *et al.*, 2016 ▸; Andrews *et al.*, 2017 ▸; Haas *et al.*, 2018 ▸). Distributions of anisotropic precipitates and a large grain size can also make difficult the interpretation of such measurements, as the intensity of the diffraction peaks depends on the orientation of the precipitates.

This paper presents a methodology to improve the signals for SAXS and WAXS characterization of metallic alloys containing anisotropic precipitates with a large grain size by rotating the samples during measurement. We will show that applying this methodology significantly improves the reliability and repeatability of the measurement of the small dimension of elongated precipitates and of their volume fraction, which will be compared with independent measurements. Two aluminium alloys from the 2*xxx* series are used for demonstration.

## 2. Samples and measuring conditions

### 2.1. Aluminium alloys of the study and their nano-precipitates

Two different materials, namely the 2618A alloy and the 2219 alloy, have been used to develop and illustrate this methodology. These alloys are classically used in aerospace applications for structural parts. The compositions of these materials are given in Table 1 ▸. These alloys were received in the T8 state prior to additional ageing.

In the 2219 alloy, the θ′ (Al_2_Cu) precipitates are known to control, through their size, morphology and volume fraction, the mechanical properties at the peak-aged state and during over-ageing (Chen *et al.*, 2009 ▸; Prabhu, 2017 ▸; Bello *et al.*, 2021 ▸). These precipitates have a platelet morphology, with a habit plane lying on the {100}_α_ planes.

The nano-precipitates which control precipitation hardening in the 2618A alloy are from the S-Al_2_CuMg family (Wang *et al.*, 2008 ▸; Elgallad *et al.*, 2014 ▸). They form as rods/laths oriented along the 〈100〉_α_ directions. Extensive studies of the precipitation sequence and the different variants of the S family showed very subtle differences between S and S′ (or S_1_ and S_2_). The main variation observed is a slight change in morphology (rod to lath with a moderate cross-sectional aspect ratio) or in lattice parameter, but with the same crystallographic structure and same composition (Gupta *et al.*, 1987 ▸; Kovarik *et al.*, 2006 ▸; Styles *et al.*, 2012 ▸, 2015 ▸). In this paper, no distinction will be made between these phases; as SAXS/WAXS measurements of both these elongated shapes are still well described by the rod model used, all of the variants will be referred to as S-phase (Al_2_CuMg).

### 2.2. Instruments used for characterization

SAXS and WAXS measurements were conducted on the BM02-D2AM beamline at the European Synchrotron ESRF (Grenoble, France) with the beam size set to 100 × 100 µm. A 2D photon-counting detector (IMXPAD D5) was set 3.5 m from the samples, while a 2D WAXS detector (IMXPAD WOS) was placed at a distance of 15 cm. Both detectors are composed of several stacked modules separated by ‘blind’ zones to give space for electronic wiring (Chahine *et al.*, 2019 ▸). This has an influence on the integration step that will be discussed later in the paper. The beam energy was 18 keV, allowing *q* ranges from 0.004 to 0.3 Å^−1^ for SAXS and from 0.75 to 4.9 Å^−1^ for WAXS. Concerning WAXS measurements, a partially transparent mask was placed for angles greater than or equal to the first peak (111) of the aluminium matrix to protect the detector from its intensity; its presence is accounted for in the data analysis. The WAXS detector has a hole in its centre to allow simultaneous SAXS/WAXS measurements. Between the two detectors, the scattered beam passed through a vacuum chamber.

In order to provide a means of comparison with the SAXS/WAXS measurements, TEM observations were also performed. A JEOL 2100F instrument equipped with a field emission gun and operated at 200 kV was used for observations. The TEM samples were prepared from thin discs and electropolished using a solution of 33% nitric acid with 66% methanol at 253 K. Observations were carried out in bright-field scanning TEM mode.

Optical microscopy was also carried out to measure grain sizes. The samples were observed with polarized light after anodic oxidation using Barker’s reagent (H_2_O–HBF_4_).

Complementary atom probe tomography was performed using a LEAP 6000 XR instrument. The acquisition was performed at 50 K, in a voltage plus laser mode, with a voltage pulse fraction of 20%, a pulse frequency of 200 Hz and a laser power of 40 pJ.

## 3. SAXS anisotropic signal in the aluminium samples

**Figure 1:**
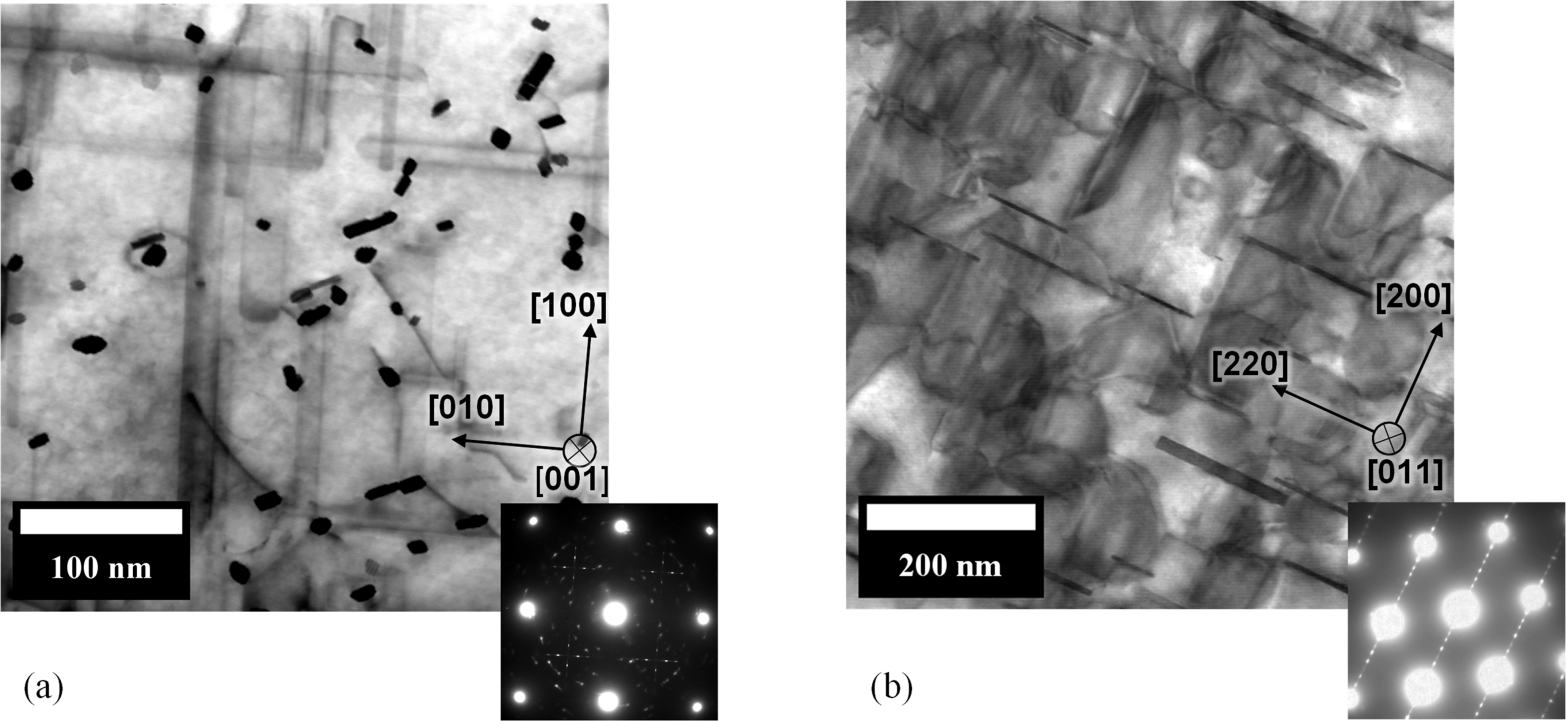
Scanning TEM bright-field images, (*a*) of the 2618A alloy aged for 2000 h at 473 K in the [001]_Al_ zone axis and (*b*) of the 2219 alloy aged for 2000 h at 473 K in the [011]_Al_ zone axis.

Before the SAXS data acquisitions, the two alloys were heat treated from their as-delivered state (T8) for 2000 h at 473 K. This allows coarsening of the hardening precipitates and ensures that a significant signal can be seen within the chosen *q* range. TEM images of hardening precipitates present in both alloys are shown in Fig. 1 ▸. In Fig. 1 ▸(*a*) the 2618A alloy is shown. The three orientations of the S rods (one normal and two others parallel to the plane of observation) can be seen. In Fig. 1 ▸(*b*), for the 2219 alloy, only one of the three orientations of θ′ platelets is visible edge on.

**Figure 2:**
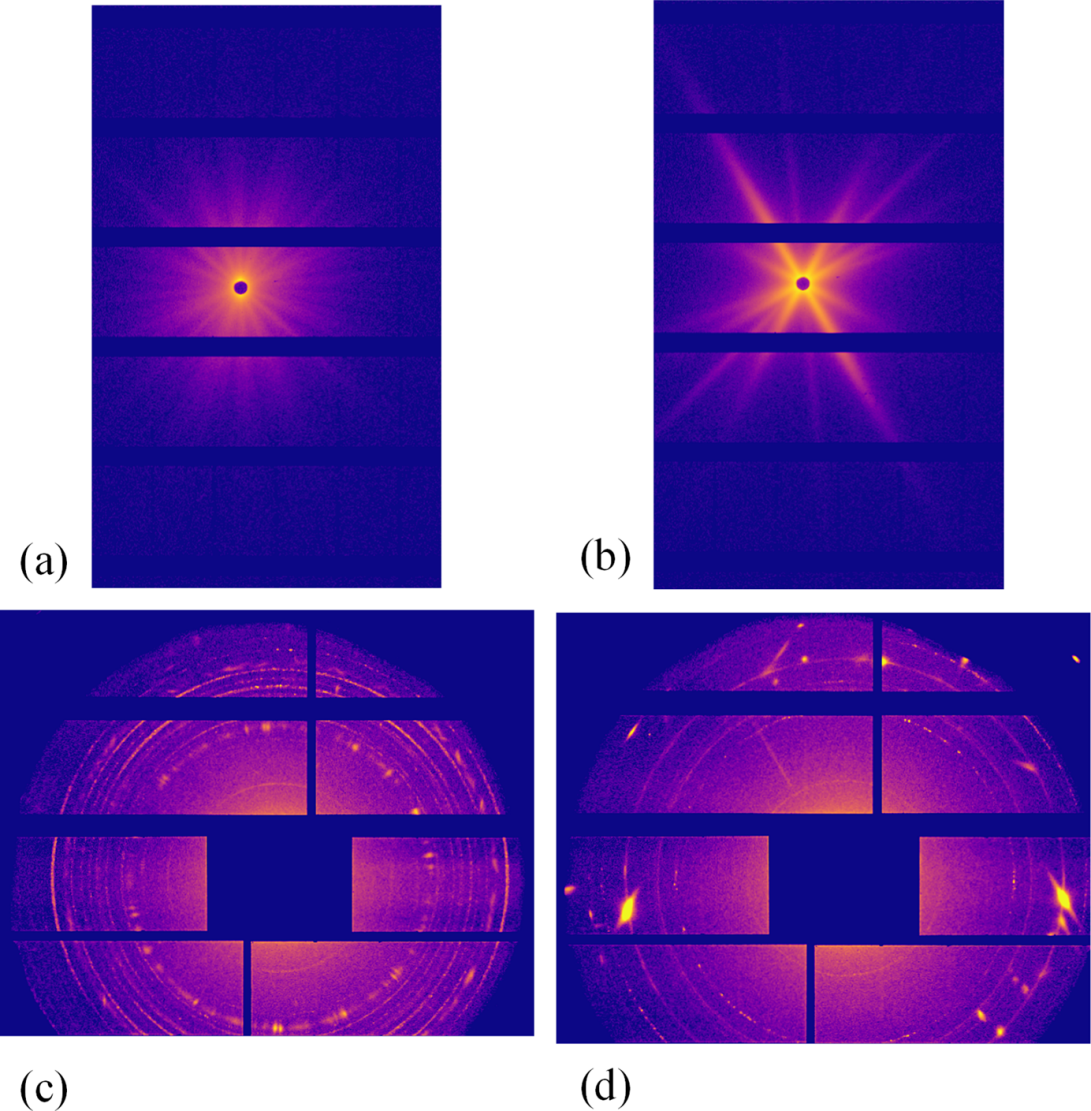
(Top row) Examples of raw SAXS images obtained for (*a*) 2618A and (*b*) 2219. (Bottom row) Examples of raw WAXS images obtained for (*c*) 2618A and (*d*) 2219.

**Figure 3:**
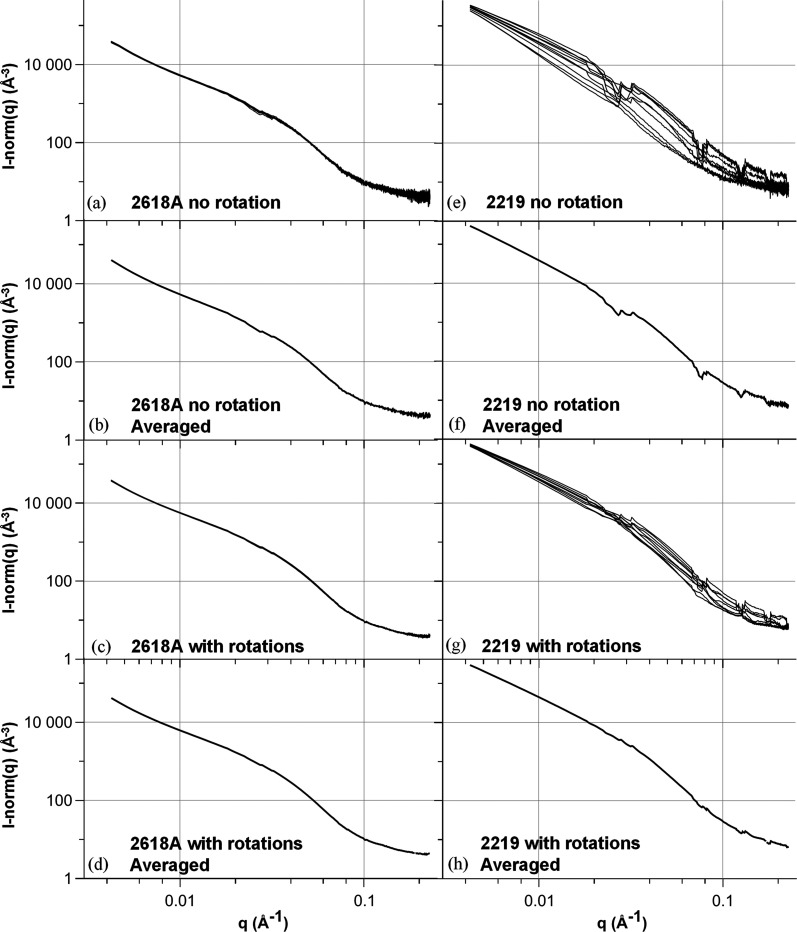
SAXS plots of the ten positions with 0° angle for (*a*) 2618A and (*e*) 2219, averaged plots of the ten positions with 0° angle for (*b*) 2618A and (*f*) 2219, averaged plots over the 30 angles for each position for (*c*) 2618A and (*g*) 2219, and averaged plots over the 30 angles and ten positions for (*d*) 2618A and (*h*) 2219. Intensity steps are observed in the *I*(*q*) plots for the 2219 alloy, even after sample rotation, indicating interactions between the anisotropic signal and the masked bands of the detector.

**Figure 4:**
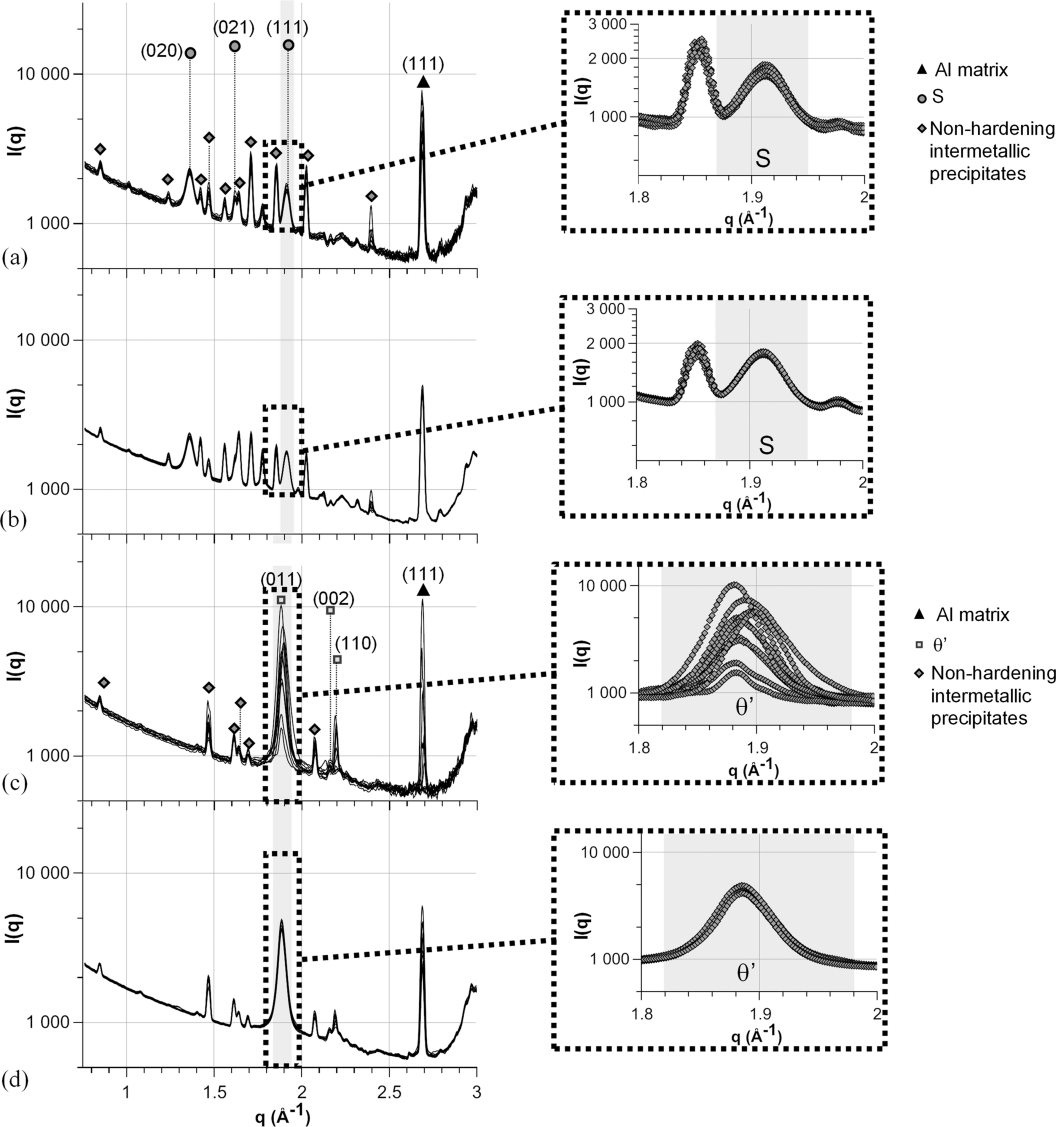
WAXS plots of the ten positions, with the enhancement of the peak related to the precipitates investigated for a 0° angle with peaks identified for (*a*) 2618A and (*c*) 2219, and after averaging along the 30 angles for each position for (*b*) 2618A and (*d*) 2219.

For each alloy, a 1 cm long and 5 mm wide SAXS/WAXS sample was prepared so that ten measurements separated by 0.5 mm along the sample’s long dimension could be performed. The sample thickness was 530 µm for the 2618A and 350 µm for the 2219 alloy because of their different compositions. More details on the thickness choice will follow. Examples of the raw images obtained for both alloys are shown in Fig. 2 ▸. The ten different raw images obtained for each alloy are displayed in the supporting information, Section S1. From these raw images, *I*(*q*) plots are obtained using the Python package *pyFAI* (Ashiotis *et al.*, 2015 ▸), which performs the azimuthal integration while masking the dead bands of the detector. The *I*(*q*) curves for the ten positions were plotted for SAXS [Figs. 3 ▸(*a*) and 3 ▸(*e*)] and WAXS [Figs. 4 ▸(*a*) and 4 ▸(*c*)]. All the SAXS *I*(*q*) plots represent the intensity normalized by the scattering cross section of an electron and the sample thickness.

The scattering signal of the 2618A alloy is close to being isotropic on the raw SAXS and WAXS images: many individual streaks, corresponding to different precipitate orientations, are visible on the SAXS images and almost continuous diffraction rings are displayed on the WAXS images [Figs. 2 ▸(*a*) and 2 ▸(*b*)]. As a consequence, after azimuthal integration the *I*(*q*) plots do not show significant discontinuity [Fig. 3 ▸(*a*)]. Moreover, little variation is observed on the *I*(*q*) plots between the ten measurement positions for both SAXS and WAXS, showing that a reliable measurement can be obtained on one individual measurement point [Figs. 3 ▸(*a*) and 4 ▸(*a*)].

In comparison, the 2219 alloy exhibits a lower number of intense streaks on the SAXS raw images [Fig. 2 ▸(*c*)]. This streaking, combined with the masked dead bands of the detector, creates abrupt troughs in the azimuthally integrated *I*(*q*) plots, as apparent in Figs. 3 ▸(*e*), 3 ▸(*f*) and 3 ▸(*g*). More importantly, a large variability along the different positions is observed for both SAXS and WAXS acquisitions (even in regions of *q* not affected by the dead bands of the detector). This large variability observed on the *I*(*q*) integrated curves can be related to the disparity of the streaks present in each image (see the supporting information) depending on the exact orientation of the grains illuminated by the X-ray beam at each position. Consequently, the SAXS results of an individual position cannot be used in the 2219 alloy to determine reliably the morphological characteristics and volume fraction of the precipitates by fitting *I*(*q*) with an appropriate model. Even after averaging over the ten positions, the SAXS *I*(*q*) plot for 2219 displays steps [Fig. 3 ▸(*f*)]. Most importantly, given the high spatial variability of the SAXS signal, it is difficult to be confident that spatial averaging would suffice to obtain a reliable measurement, since this type of alloy shows a relatively strong crystallographic texture. Several approaches can be employed to mitigate the effects of masked bands, such as acquiring the signal at two different detector positions or using machine learning algorithms to patch the gaps in the detector (Masto *et al.*, 2024 ▸). However, these methods do not address the underlying anisotropic nature of the signal, which leads to overall intensity variations.

Regarding WAXS measurements, the situation is actually less favourable than for SAXS. The intensity variability is made even higher because the precipitate’s Bragg peaks do not necessarily intersect with the detector, in contrast to the SAXS geometry which always includes the origin of reciprocal space. Moreover, simultaneous SAXS/WAXS geometry generally has strong limitations in angular range and thus the number of measured peaks. This, combined with the small size and volume fraction of precipitates, explains why a full Rietveld refinement of the precipitate diffraction signal cannot be achieved and rather a semi-quantitative analysis is sought. In the present case, similar conclusions can be drawn, namely Fig. 4 ▸(*c*) shows that it is not possible to obtain reliable results in the 2219 alloy about the precipitation state of θ′ as the intensity of the diffraction peaks related to these precipitates varies too much from one measuring position to another. In contrast, the WAXS signal from the 2618A alloy shows little variability between the positions and a clear appreciation of the precipitation state can be obtained [Fig. 4 ▸(*a*)].

The differences exhibited by the two alloys can be explained by a variety of factors. Firstly, the degree of anisotropy of the SAXS signal depends on the morphology of the precipitate’s Fourier transform as the measured signal corresponds to a cross section of the Ewald sphere. The Fourier transform (FT) of the θ′ platelets present in the 2219 alloy is a thin peak elongated in the direction normal to the plane of the platelet, whereas the FT of S rods has a pancake shape whose short axis lies in the direction of the rod (platelet-like morphology). Therefore, there is a higher variability in the cross sections obtained and measured by the SAXS detector for θ′ platelets than for S rods. An illustration of the Ewald sphere for both cases is given in Section S3 in the supporting information.

**Figure 5:**
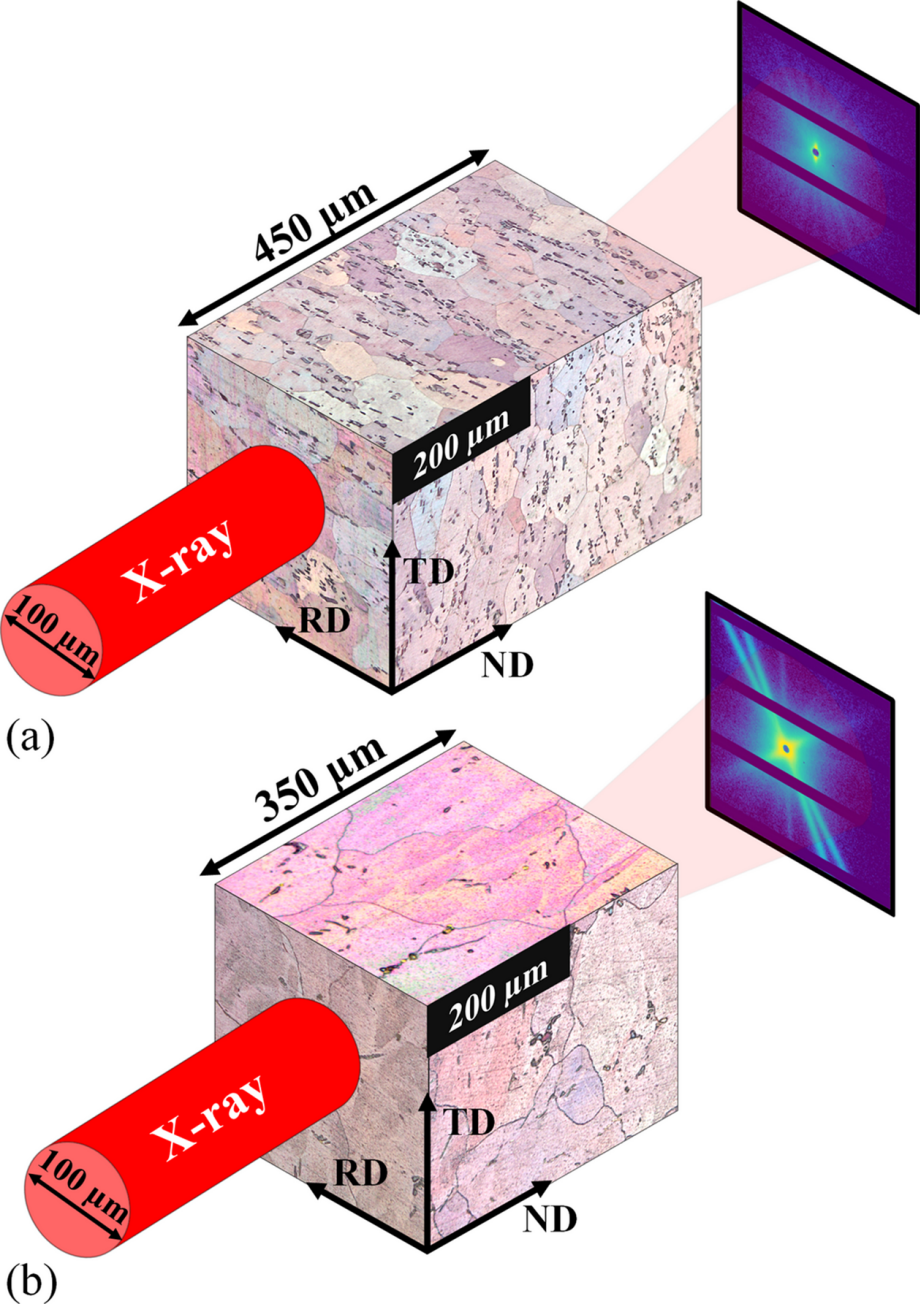
An illustration of the influence of grain size and sample thickness on the number of grains interacting with the X-ray beam for (*a*) 2618A and (*b*) 2219.

Secondly, the orientation distribution within the volume illuminated by the X-ray beam is far from random. This distribution depends both on the number of variants per grain and on the number of grains in the illuminated volume. There are three θ′ and S orientations possible for each grain orientation. The grain size has been evaluated by optical microscopy (Table 2 ▸ and Fig. 5 ▸). The 2219 alloy exhibits a larger grain size in all directions than the 2618A alloy. Considering the beam size (100 × 100 µm), grain size and sample thickness, the illuminated volume for the 2219 alloy corresponds to the volume of 0.4 of a grain, meaning a low number of grains will be responsible for the scattered signal. This limits the number of orientations of the precipitate and explains the low number and high variability of the measured precipitate streaks. Conversely, for the 2618A alloy, the irradiated volume corresponds to the volume of approximately 18 grains, allowing more orientations and hence leading to a better average and thus a more isotropic SAXS/WAXS signal.

## 4. Rotations to obtain isotropic results from anisotropic samples

In order to improve the reliability of the SAXS/WAXS results, tilt rotation with the rotation axis lying in the short direction of the samples was carried out to obtain a variety of precipitate orientations for a given position, allowing averaging of the *I*(*q*) plots along the rotation angles.

### 4.1. Experimental setup for rotating samples

Implementation of a rotation motor in the experimental assembly has to fulfil several conditions, the most important being that the rotation axis must coincide with the beam axis. Furthermore, the distance between the SAXS/WAXS detectors and the sample must remain constant during rotation and translation from one position to another on the sample tray (allowing space-resolved measurements). No fewer than four translations are required to meet these conditions.

**Figure 6:**
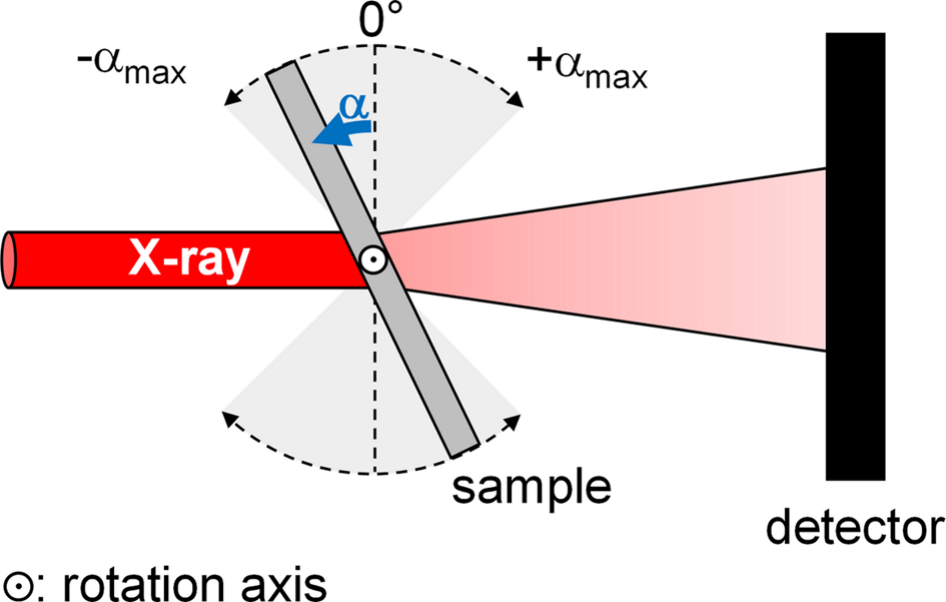
A schematic diagram of the rotation setup viewed from above.

Three orthogonal translation stages are positioned above a vertical rotation axis, with an additional translation stage below to align the rotation axis with the X-ray beam (Fig. 6 ▸). Two translations are used to scan the sample tray while the third one ensures a constant sample-to-detector distance. To maintain proper sample positioning during measurement and rotations, using a fixed camera is at least helpful if not necessary. The definition of the centre of rotation was made using this camera placed above the experimental setup. This allows aligning the rotation axis constantly in the beam axis and computing the adequate compensation for the *Y* translation to fix the sample-to-detector distance. This becomes particularly important when scanning long samples. Further details on the positions of the different stages are available in Section S4 in the supporting information.

Concerning the samples, the thickness can be optimized to minimize the effect of the variation in the thickness and transmission on the variation in scattered intensity at all rotation angles. The total scattered intensity is proportional to the sample thickness *t* multiplied by the transmission Tr,
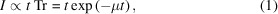
μ being the linear absorption coefficient of the material, which depends on the beam energy and the alloy composition (μ_2618A, 18 keV_ = 16.9 cm^−1^ and μ_2219, 18 keV_ = 19.2 cm^−1^).

**Figure 7:**
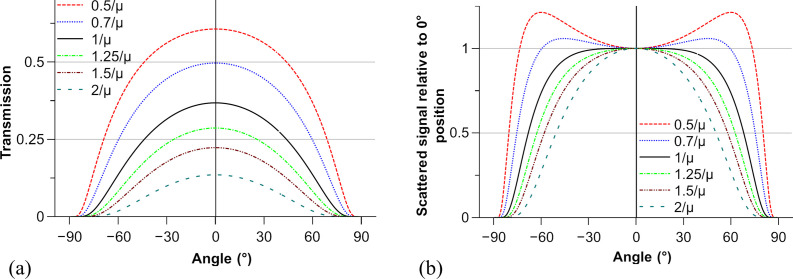
The theoretical variation of (*a*) the transmission and (*b*) the relative signal as a function of the rotation angle and the sample thickness.

As the effective sample thickness varies during rotation, assuming the sample is infinitely long in the transverse direction we have
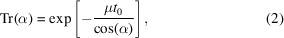
α being the angle between the beam and the normal to the sample and *t*_0_ the sample thickness for a 0° angle. Consequently,
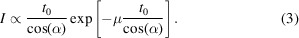
Usually, the sample thickness is chosen to get the maximum signal (thus at 1/μ). Here, the aim is to minimize the variation in the scattered intensity with rotation. To do so, we calculate in Fig. 7 ▸ the variation in (*a*) the transmission and (*b*) the relative signal (compared with the 0° angle) as a function of the sample-to-beam angle (α) and the sample thickness (*t*_0_).

To obtain a signal intensity that varies the least for a −60°/60° range, the optimal sample thickness should be equal to 0.7 times the usual optimal sample thickness. Low overall intensity variability could allow continuous measurement during rotation without a strong need for angle-dependent signal normalization. Different methods can be used for the acquisition during the rotation of the sample. In this paper, semi-continuous acquisition was used, meaning that during rotation numerous SAXS/WAXS acquisitions were made, each one on a defined angular range (here 3°). Normalization was performed for each measurement separately. A fully continuous approach (corresponding to a single acquisition during the full rotation) can be used if the relative signal variability is negligible over the scanned angular range. The appropriateness of these acquisition methods will be discussed in Section 4.3.

### 4.2. Results obtained for aluminium alloys using rotations

#### 4.2.1. Comparison between the two alloys

In this experiment, the sample was rotated from −45° to 45°. SAXS/WAXS acquisitions were made from the same ten positions as in Section 3. The measurement time was optimized by performing acquisitions during motor sample rotation from −45° to 45° and *vice versa*. A compromise between rotation speed and acquisition time had to be fixed to keep a reasonable measuring time with sufficient signal intensity. Here, semi-continuous measurements during rotation were acquired with a 3° s^−1^ rotation speed and a one-second acquisition, giving 30 images per position with each image corresponding to a 3° rotation. Therefore, a total of 300 *I*(*q*) plots per alloy were obtained. No measurements were performed during acceleration and deceleration as the rotation speed needs to be constant. The effective angular range was then −42.6° to 42.6°. Examples of the raw images for each 3° rotation compared with the image averaged along all rotation angles for SAXS and WAXS are given in Sections S5 and S6 in the supporting information.

**Figure 8:**
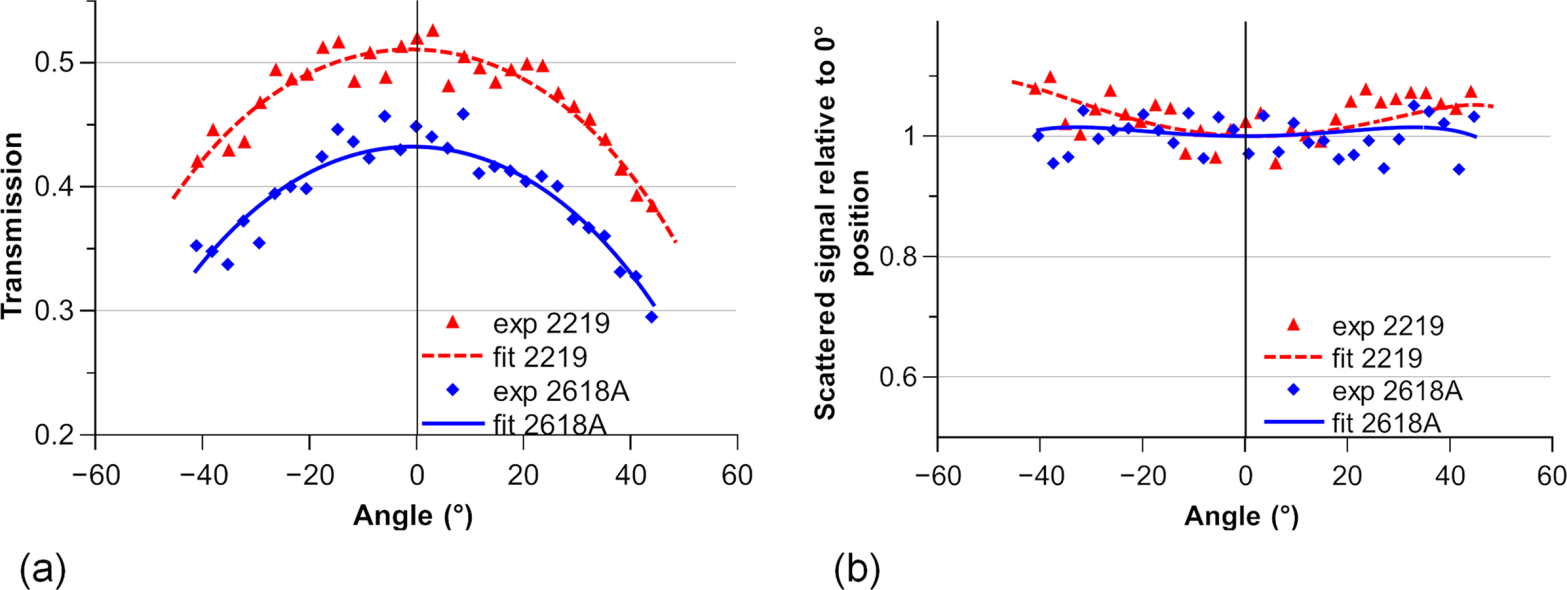
(*a*) Measured transmission and fitted transmission for both alloys along the angles for a given position. (*b*) Measured and fitted relative SAXS signal compared with that at the 0° angle for the same position along the angles.

The measured transmission and relative scattered intensities are plotted in Fig. 8 ▸ and fitted to estimate the uncertainty and to compare different normalization methods. To evaluate the evolution of the SAXS signal, we calculate −ln(Tr)Tr/μ (Tr being the transmission), which is equal to the sample thickness multiplied by the transmission according to equation (1) and is proportional to the scattered intensity [equation (2)]. We then normalize this value by the corresponding value at a 0° angle to obtain the plot in Fig. 8 ▸(*b*). Conventional normalization using the measured transmission for each angle was compared with a fitted normalization defined using the angle-dependent transmission evolution.

Fitting the transmission measurements is done by fitting *t*_0_ in equation (2). The sample thicknesses given by fitting the measured transmission (547 and 336 µm) are in good agreement with the thicknesses measured by touch probe (530 and 350 µm).

Along the full rotation range, the normalized theoretical scattered intensities only differ by 0.4% on average and by a maximum of 2.4% for the acquisitions from 2219. For 2618A, the two intensities differ by 0.2% on average with a maximum of 0.8%. Thus, a low effect of the uncertainty on the transmission measurement is to be expected.

**Figure 9:**
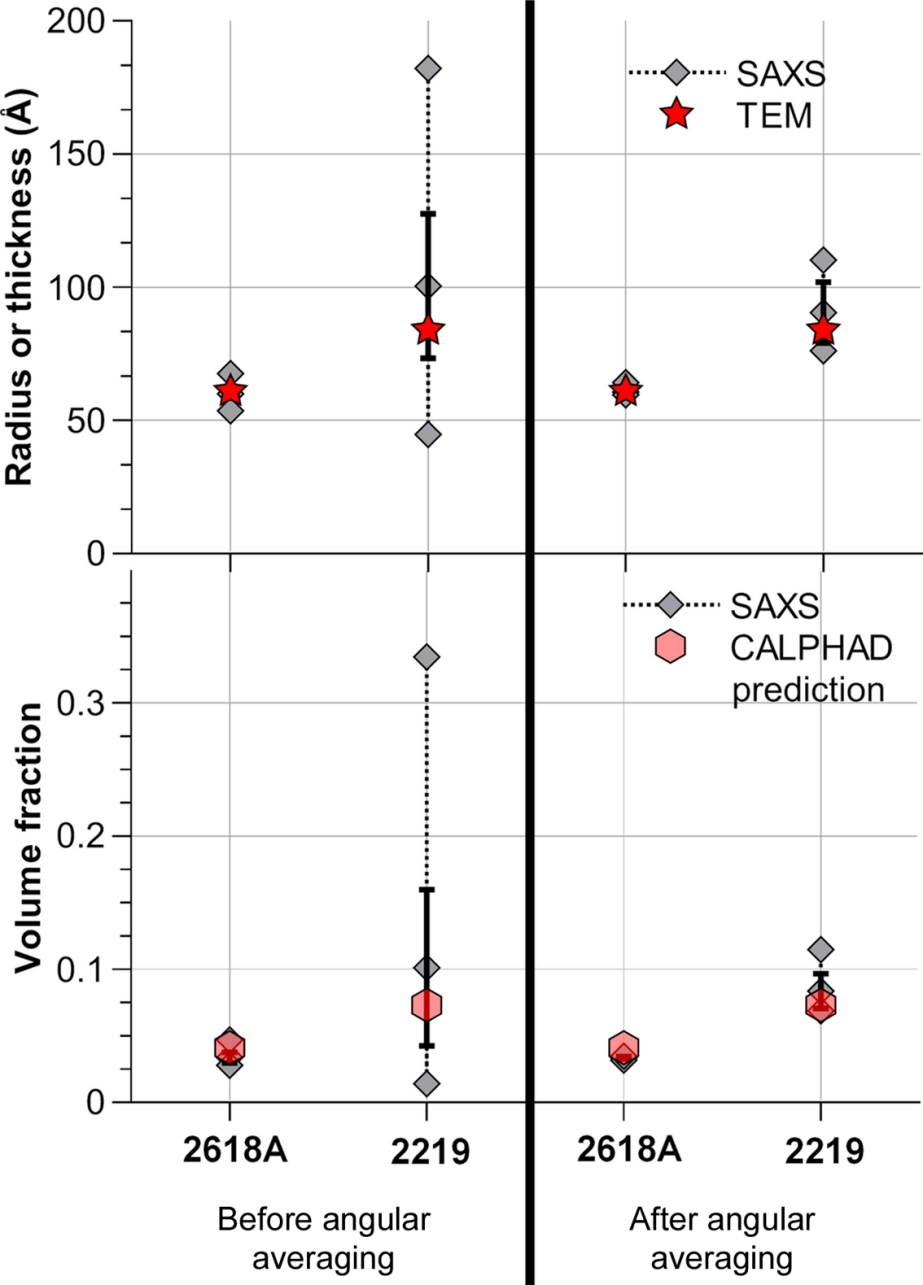
Variability of the SAXS fit results (mean precipitate thickness/radius and volume fraction) before and after angular averaging for the samples of 2618A and 2219 aged for 2000 h at 473 K. For SAXS results, the minimum, maximum and mean values are represented with the standard deviation plotted on the mean value as error bars. Size measurements are compared to TEM observations and volume fractions are compared with CALPHAD equilibrium predictions.

Given the large size distribution combined with the slight variability of shapes, each obtained SAXS curve was fitted by a semi-empirical model (Hammouda, 2010 ▸) corresponding to flat particles (for the 2219 alloy) and long particles (for the 2618A alloy). Given the size of the elongated precipitates provided by TEM measurements, the measurement of the precipitate long dimensions (several hundreds of nanometres) would require an ultra-small-angle setup, which is out of the current scope of the study. Additionally, the smaller *q* range could be affected by various contributions that are challenging to separate, including reflectivity (Kühnhammer *et al.*, 2022 ▸; Lu *et al.*, 2022 ▸) or features inherent to the alloy, such as grain boundaries and precipitates at these boundaries. Therefore, the aim here is to provide a reliable measurement of the precipitate small dimension and volume fraction, which are in any case the most important parameters for controlling the material’s mechanical properties. For such precipitate morphologies, the SAXS signal is separated into three regions depending on the *q* range: a Porod behaviour in *q*^−4^ at high *q*, a low-*q* Guinier region, and a Porod-type behaviour in *q*^−2^ (for platelets) or *q*^−1^ (for rods) in the intermediate *q* range. The coefficient for the intermediate range can be expressed as a function of the contrast, the volume fraction and the small dimension only (Hammouda, 2010 ▸). Within the chosen *q* range, only the high-*q* and mid-*q* ranges of the precipitate signal are measured. Therefore, the smallest dimensions (thickness of the platelets for the 2219 alloy and radius of the rods for the 2618A alloy) are determined along with the volume fraction. The variability of the fit parameters for both alloys is shown in Fig. 9 ▸ for the ten measurements made along the sample, both for classical measurements made at fixed sample orientation and after rotational averaging. These results are compared with independent measurements made on TEM micrographs for precipitate radius/thickness measurement. Details of the TEM measurements are available in Appendix *A*[App appa]. Concerning the volume fraction measurements, given the late stage of ageing of these alloys it can be expected that the precipitate volume fraction is very close to the equilibrium value. Therefore, the SAXS-determined values are compared with CALPHAD equilibrium values obtained using the TCAL9 database of *Thermo-Calc* (Andersson *et al.*, 2002 ▸); this hypothesis will be confirmed in the next section by an atom probe tomography (APT) measurement.

Before angular averaging, the fitted parameters vary only slightly over the 300 plots for the 2618A alloy, and they are in good agreement with TEM observations regarding the radius of the precipitates. A variability of 10% to 20% is still observed for volume fraction fitting. For the 2219 alloy, significant variations are observed with volume fractions ranging from 0.01 to 0.34, the latter being inconsistent given that the volume fraction cannot be more than 0.09 on the basis of the overall alloy composition and precipitate composition (Al_2_Cu). Precipitate thickness measurements also display a high variability, and no reliable or indicative measurement can be obtained with a single measurement.

For each position, *I*(*q*) plots were averaged on the 30 angles acquired after normalization of each plot using the measured transmission for each angle [Figs. 3 ▸(*c*) and 3 ▸(*g*)]. The resulting plots were then fitted, providing ten values of fit parameters (precipitate radius/thickness, volume fraction) which are also presented in Fig. 9 ▸. A clear improvement can be seen for both alloys, with a greater enhancement for 2219. After averaging, fit parameters for this alloy vary by a maximum of 20% of the mean value. The mean fitted thickness (90 Å) is reasonably close to the mean thickness measured by TEM (84 Å); the uncertainty remains significant, albeit evidently lower than the 100–200% variability before averaging. The plots averaged along the ten positions and 30 angles correspond to a close to isotropic SAXS signal [Figs. 3 ▸(*d*) and 3 ▸(*h*)]. Concerning the 2618A alloy, Fig. 9 ▸ also shows an improvement in the measurement variability for both the volume fraction and radius of S precipitates, even though the situation without averaging was already satisfactory. In both alloys, not only is the spatial variability of the measured volume fraction improved by the rotation, but the absolute value converges as expected to that of the CALPHAD predictions.

The same averaging was performed for the WAXS data [Figs. 4 ▸(*b*) and 4 ▸(*d*)]. Similarly to the case of SAXS, the plot’s repeatability for the 2618A alloy is further improved by this averaging, and a clear improvement is visible on the 2219 plots (where the measurements without rotation were useless), with a close to constant peak intensity for θ′ precipitates from one position to another. This opens the possibility of using WAXS in combination with SAXS *e.g.* to evaluate semi-quantitatively the occurrence of a phase transition or a change in lattice parameter.

#### 4.2.2. Characterization of the 2219 alloy during ageing

**Figure 10:**
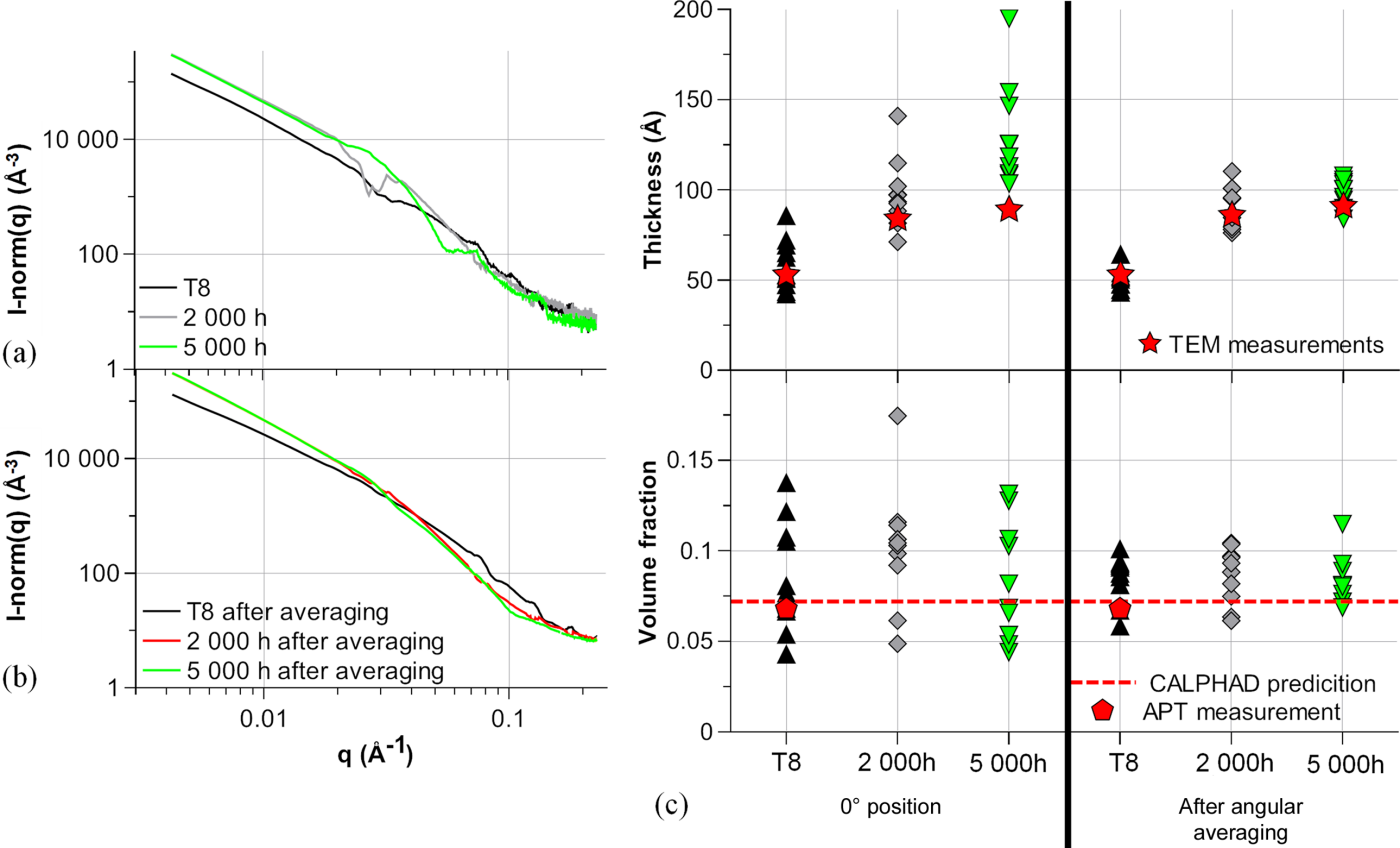
(*a*) SAXS plots for the different ageing times at 0° angle compared with (*b*) averaging along angles. (*c*) The resulting fitted parameters (compared with TEM measurements for precipitate thickness, and CALPHAD equilibrium predictions and APT measurement for volume fraction).

The 2219 specimen aged for 2000 h at 473 K was compared with a specimen in the initial state (T8) and a specimen aged for 5000 h at 473 K. SAXS/WAXS results were obtained under the same conditions with and without rotation of the samples. Fig. 10 ▸ shows the different SAXS plots for a single position and the resulting fitted parameters for the different positions.

Morphological results are compared with TEM observations which are detailed in Appendix *A*[App appa]. In the case of the T8 temper, we also acquired APT data. As discussed in the *Introduction*[Sec sec1], such data are less appropriate than TEM for determining the precipitate dimensions reliably or the volume fraction directly. However, using the distribution of isolated atoms for the determination of the matrix composition [the DIAM method, proposed by De Geuser & Lefebvre (2011 ▸)] gives a very precise measurement of the aluminium matrix solute content. Since the precipitate composition (Al_2_Cu) and molar volume are known, this solute content can be converted into a precipitate volume fraction. The details are provided in Appendix *B*[App appb] and the resulting volume fraction is plotted alongside the CALPHAD prediction in Fig. 10 ▸(*c*).

Angular averaging reduces the spread and gives results in better agreement with the TEM observations. In this particular case, the evolution of precipitate size would be overestimated without the use of rotation and the evolution of volume fraction could not be estimated because of the significant spread. Applying the sample rotation enables a drastic reduction in the spread in precipitate measurements (both size and volume fraction) and enables a clear determination of the evolution of these parameters with ageing time.

**Figure 11:**
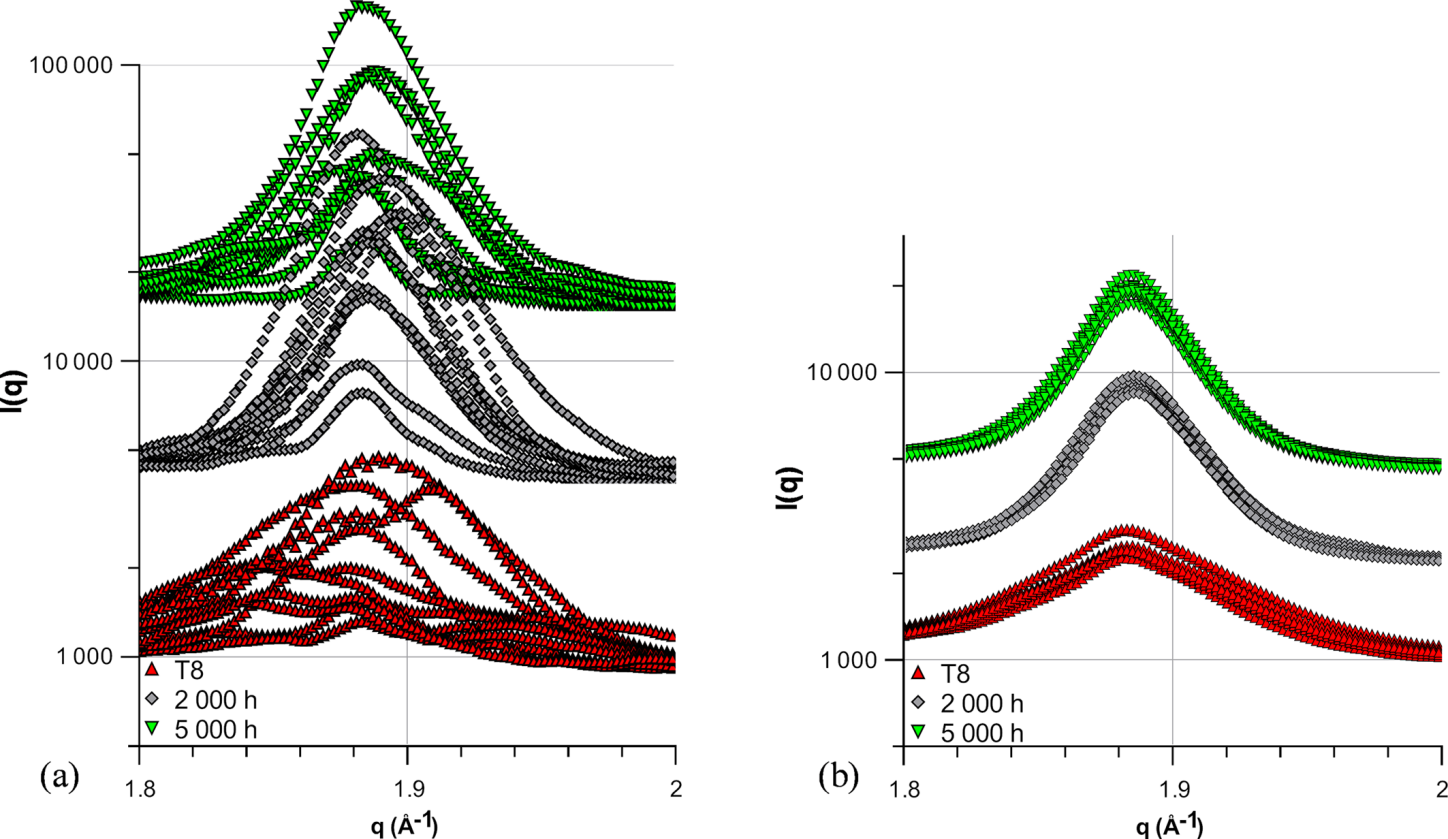
Evolution of the θ′ peak on the WAXS plots, (*a*) for the different ageing times at 0° angle and (*b*) after angular averaging. The results of the different ageing conditions are offset for comparison.

WAXS results are displayed in Fig. 11 ▸. Without rotations, the results are highly position dependent and no conclusions can be drawn from the different plots.

Angular averaging allows comparison between the different precipitation states. A clear narrowing of the θ′ peak is visible between the T8 state and the sample aged for 2000 h at 473 K, while a low evolution between 2000 and 5000 h is visible. Simultaneously, the area below the peak is close to constant between the different states. These results are in good agreement with the SAXS and TEM results, showing a clear size evolution mainly between the T8 state and the sample aged for 2000 h, while the volume fraction remains virtually constant throughout the ageing process.

### 4.3. Guidelines for a time-efficient and reliable experiment with a rotation setup

**Figure 12:**
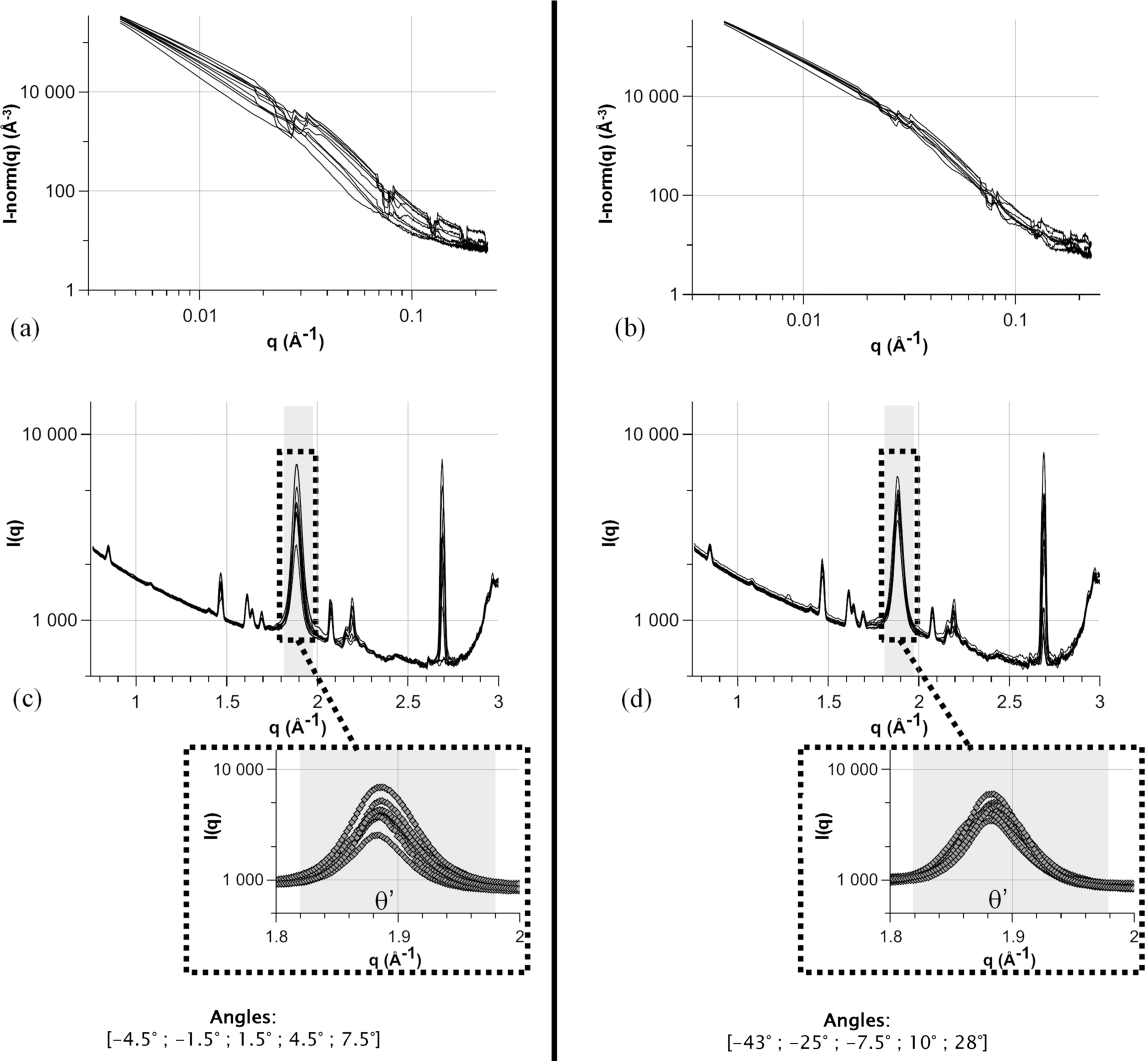
A comparison of two averaging methods for the 2219 alloy aged for 2000 h. Plots of the ten positions after averaging over five consecutive angles for (*a*) SAXS and (*c*) WAXS versus averaging over five widely dispersed angles for (*b*) SAXS and (*d*) WAXS.

Section 4.2 presents an example of the use of a rotation methodology to improve measurement reliability in the case of non-isotropic signals, but parameters can be optimized depending on the constraints and the material observed. Various compromises need to be found to maximize the time efficiency and reliability of the measurements. To limit the experimental time, different strategies can be adopted. Limiting the number of angular positions or the global angular range is one of them. Fig. 12 ▸ shows a comparison between two approaches where only five of the 30 angular positions are used to obtain the averaged SAXS/WAXS plots for each of the ten positions for the 2219 alloy aged for 2000 h. It appears that choosing widely spread angles results in a better averaging of the SAXS and WAXS plots. This means that the biggest angular range should be used to obtain the best averaging. In the case of spatially resolved measurements (*e.g.* when studying microstructures with microstructural gradients), the spatial uncertainty induced by the rotation of the sample needs to be kept in mind. If possible, choosing the rotation axis to be parallel to the microstructural gradient is the best option and this would not result in an additional uncertainty. If the rotation axis is orthogonal to the gradient, a global uncertainty of *t*_0_ tan(α_max_) on the position needs to be taken into account, with *t*_0_ the sample thickness and α_max_ the maximum angle.

**Figure 13:**
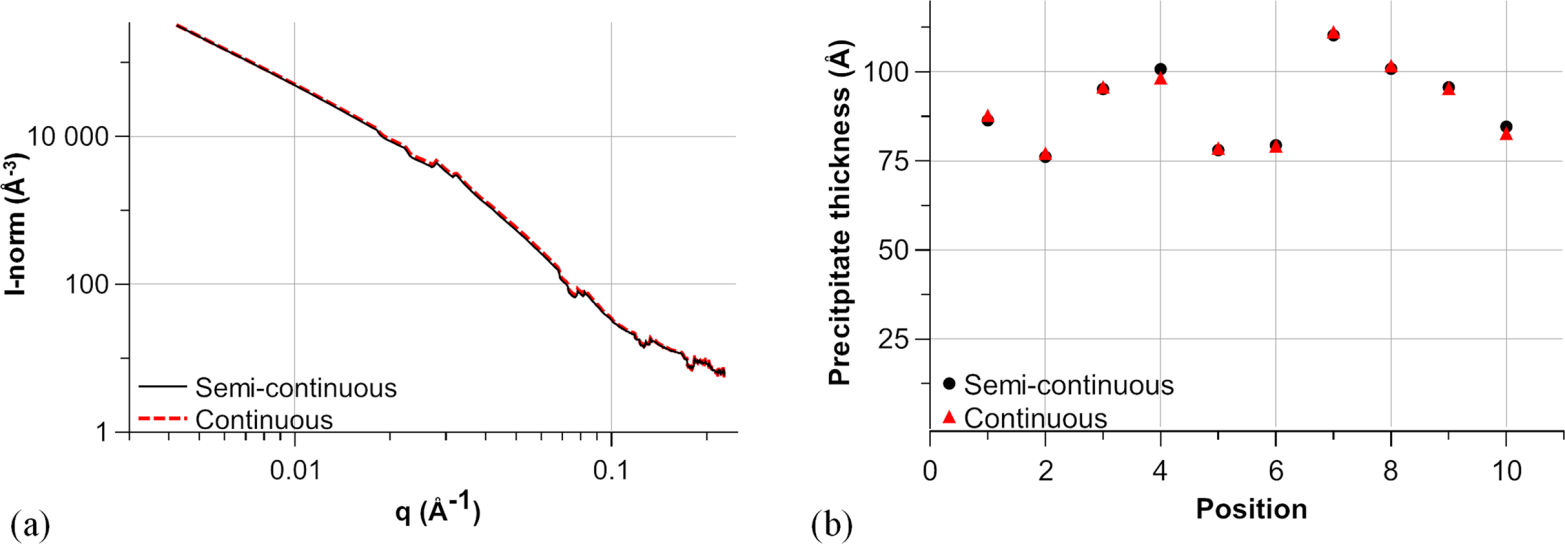
A comparison of semi-continuous and simulated continuous acquisitions for the 2219 specimen aged for 2000 h. (*a*) Normalized SAXS plots for a given position and (*b*) fitted precipitate thicknesses for the different positions.

Concerning the acquisition method, continuous acquisition as introduced in Section 4.1 can be used if the variability in the scattered intensity is low. One unique continuous measurement during full rotation of the sample could be done and just a single normalization would then be performed. Continuous measurement was simulated for the 2219 alloy by summing the various non-normalized intensities before a single normalization using the mean transmission along the angles. The resulting plot is compared with the semi-continuous case in Fig. 13 ▸(*a*).

The mean variability in the normalized scattered intensities for the ten positions is no more than 0.1%, with a maximum of 8%. For each position, the *I*(*q*) curve was fitted; the resulting fitted precipitate thicknesses are displayed in Fig. 13 ▸(*b*). Similar results are obtained for simulated continuous and semi-continuous measurements, meaning that in our case continuous measurement could have been used because of the sample thickness optimization. Using this method can save time and would also limit the data storage required, as only one image would be acquired for each position. If the scattered intensity is expected to vary a little more, semi-continuous measurements as used in our example seem suitable and may result in a similar measuring time, but with much more data storage needed. As normalization needs to be accounted for for each plot separately, the choice of angular step is limited by both the measurement noise and the variability in the scattered intensity along the angles.

Finally, in the worst-case scenario (*i.e.* if high variation is expected because the ideal sample thickness cannot practically be used), non-continuous acquisitions can still be used. Each measurement is taken for a fixed orientation and rotations take place between each acquisition. Classical normalization is computed for each orientation separately. The experimental time is then dependent on the number of orientations measured and will be longer than for continuous or semi-continuous acquisitions.

In order to improve the averaging of precipitate orientations further, several additional steps could be taken. First, two perpendicular rotation axes could be used, with the cost of a more complex setup. Alternatively, the sample geometry could be set to a cylinder of diameter equal to the attenuation length, which would allow the sample to be rotated 360°. For small attenuation lengths (a few hundreds of micrometres for Al alloys, much lower for Ti-, Ni- or Fe-based alloys), however, the sample preparation and axis rotation alignment would be much more complicated. Concerning WAXS acquisition, a more quantitative data reduction would need to account for the variability in the self-absorption of the sample. This self-absorption depends on the scattering angle, as outlined by Pauw (2013 ▸), as scattered X-rays do not cross the same effective sample thickness. Furthermore, in the context of sample rotation, the self-absorption will also be affected by the sample tilt. Consequently, the 2D data should be normalized by the effective sample thickness through which the beam passes before azimuthal integration, depending on the scattering angle, sample geometry and tilt angle. The variability in self-absorption is much less significant for SAXS data, particularly in the present alloys with low attenuation coefficients. Applying this correction could still enhance the precision of the measurements.

## 5. Conclusions

We have shown that applying sample rotations during SAXS/WAXS acquisition can lead to more reliable signals for metallic alloys with anisotropic precipitates. The setup consists of four translational stages and a rotational one, while a fixed camera is necessary to ensure good positioning of the sample. Compromises need to be found on different scales concerning the sample thickness, the intensity of the scattered signal, the angular range, the rotational speed, the experimental time, the acquisition time or even the data storage. If the sample thickness is optimized to obtain a low varying scattered intensity along the angles, continuous acquisitions during rotations can be used to save time and limit the amount of data. The maximal angular range should be chosen to obtain the best results.

This methodology can also improve spatially resolved measurements such as for materials with microstructural gradients, like welded structures. In the common case of highly textured materials, this methodology is particularly suitable, since the same level of reliability could not be obtained by only multiplying the measurement positions. Its applicability can be extended to all systems showing anisotropic precipitates such as many Al-, Mg-, Ti-, Ni- or Fe-based systems.

## Supplementary Material

Supporting Information S1-S6. DOI: 10.1107/S1600576724009294/jl5089sup1.pdf

## Figures and Tables

**Figure 14:**
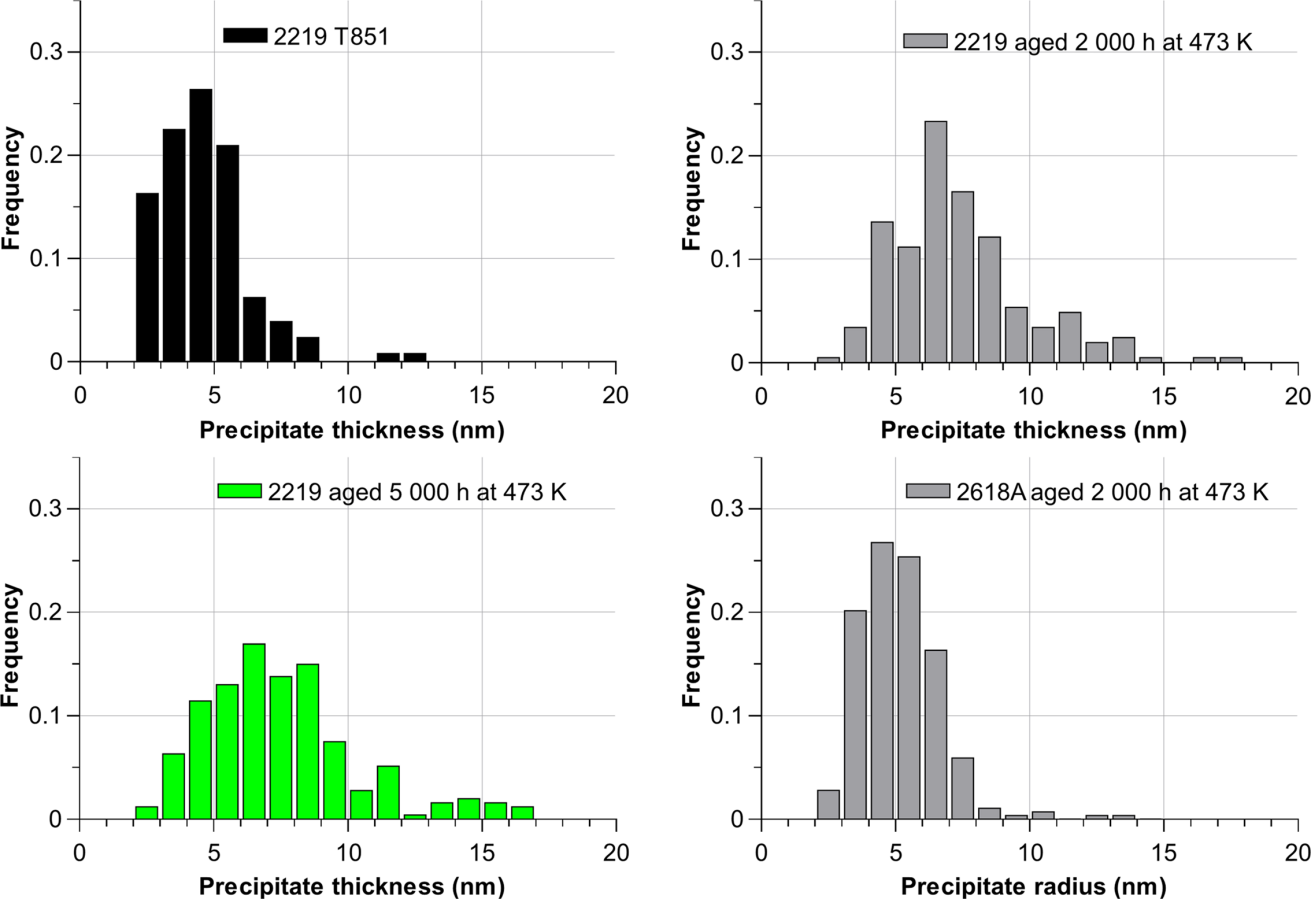
Distribution of precipitate sizes (thickness or radius) measured from TEM observations for the three ageing conditions of the 2219 alloy and the aged condition of the 2618A alloy.

**Figure 15:**
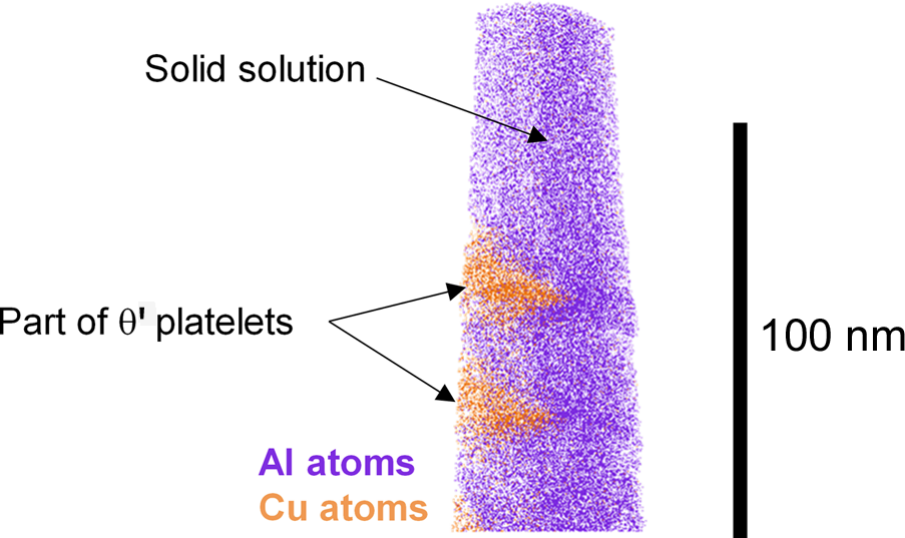
Reconstruction of the volume probed by APT.

**Table 1:** Composition (%wt) of the two aluminium alloys supplied for this study, measured by inductively coupled plasma atomic emission spectroscopy

	Al	Cu	Mn	Mg	Fe	Ni	Si	Zr	V
2618A	93.2	2.5	0.05	1.5	1.1	1.3	0.23	–	–
2219	93.1	6.2	0.27	0.007	0.1	–	0.04	0.11	0.07

**Table 2:** Grain sizes measured by optical microscopy after anodic oxidation along the rolling direction (RD), transverse direction (TD) and normal direction (ND) The main value and the standard deviation are given. A minimum of 150 grains were measured for each direction.

	RD	TD	ND
2618A	88 ± 11 µm	66 ± 3 µm	46 ± 6 µm
2219	296 ± 57 µm	199 ± 31 µm	149 ± 32 µm

**Table 3:** Parameters used to compute the volume fraction of precipitates from the APT experiment

Symbol	*c*_0_	*c*_p_	*c*_m_	Ω_p_/Ω_Al_
Value	2.7 at%	33 at%	0.26 at%	0.91

## Appendix A Method and results of TEM measurements

Precipitate size distributions are obtained for the different alloys and ageing conditions with a minimum of 200 precipitates per condition (Fig. 14 ▸). Volume-weighted averages are calculated from the TEM distributions to be compared with the SAXS results (also volume weighted). Here, platelets and rods are considered as cylinders of radius *r* and length (or thickness) *t*. For platelets *t* << *r* and for rods *r* << *t*.

For rods, the volume-weighted average radius of the distribution (*r*_m,v_) is determined as follows:

with *f*(*r*) the number density of the distribution for the radius *r*.

Therefore, in our case, for experimental TEM measurements,

with *r_i_* the radius of rod *i*, *V_i_* its volume and *n* the total number of rods measured.

Considering the volume of a cylinder and assuming that *r* << *t* with *t* constant, we then obtain

Similarly, for platelets, the volume-weighted average thickness (*t*_m,v_) of the distribution is given by

## Appendix B Method and results of APT measurement

APT acquisition was carried out on a tip from the 2219 alloy at the initial T8 state. A volume of five million atoms is shown in Fig. 15 ▸. Two ends of θ′ platelets are visible.

The volume fraction of θ′ precipitates in the 2219 alloy at the initial T8 state was computed from this experiment. The method relies on measuring the copper content in the solid solution using the DIAM method and then calculating the volume fraction by mass balance of copper in the alloy. According to CALPHAD prediction, the non-hardening precipitates consume a low level of copper (typically Al_28_Cu_4_Mn_7_ with a volume fraction of less than 1%).

Considering that copper is either in the θ′ precipitates or in the aluminium matrix, and taking into account the difference in molar volume between the aluminium matrix and the θ′ precipitates, we have

with *f*_v_ the volume fraction of the θ′ precipitates, *c*_0_ the total copper content in the APT tip, *c*_p_ the copper content in the precipitate (33%), *c*_m_ the copper content in the solid solution (calculated using the DIAM method), and Ω_p_ and Ω_Al_ the molar volumes of the θ′ precipitate and of the aluminium matrix, respectively. The values of the different variables are given in Table 3 ▸.

We then obtain a volume fraction of 6.8% of θ′ precipitates.
