# Supplementary material for: Improving the reliability of small- and wide-angle X-ray scattering measurements of anisotropic precipitates in metallic alloys using sample rotation
Source: J Appl Crystallogr. 2024 Nov 4;57(Pt 6):1800–14. doi: 10.1107/S1600576724009294 (PMC11611286; doi:10.1107/S1600576724009294)

JOURNAL OF
APPLIED
CRYSTALLOGRAPHY

Volume 57 (2024)

Supporting information for article:

Improving the reliability of small- and wide-angle X-ray scattering measurements of anisotropic precipitates in metallic alloys using sample rotation

Thomas Perrin, Gilbert A. Chahine, Stéphan Arnaud, Arthur Després, Pierre Heugue, Alexis Deschamps and Frédéric De Geuser

S1. SAXS and WAXS raw images for 10 different positions (0° rotation angle) for the 2219 alloy aged 2000 h at 473 K

S1.1. SAXS images

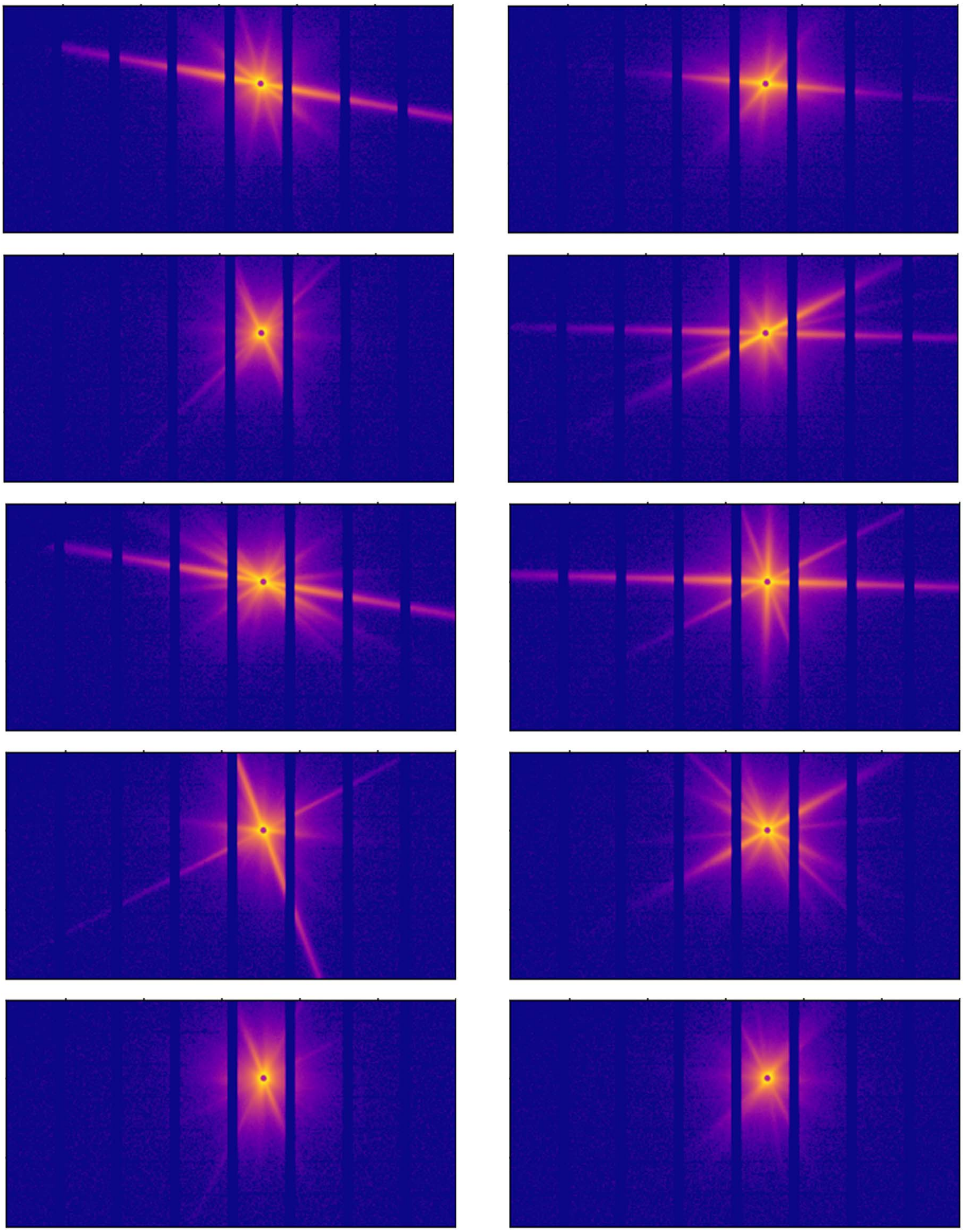

S1.2. WAXS images

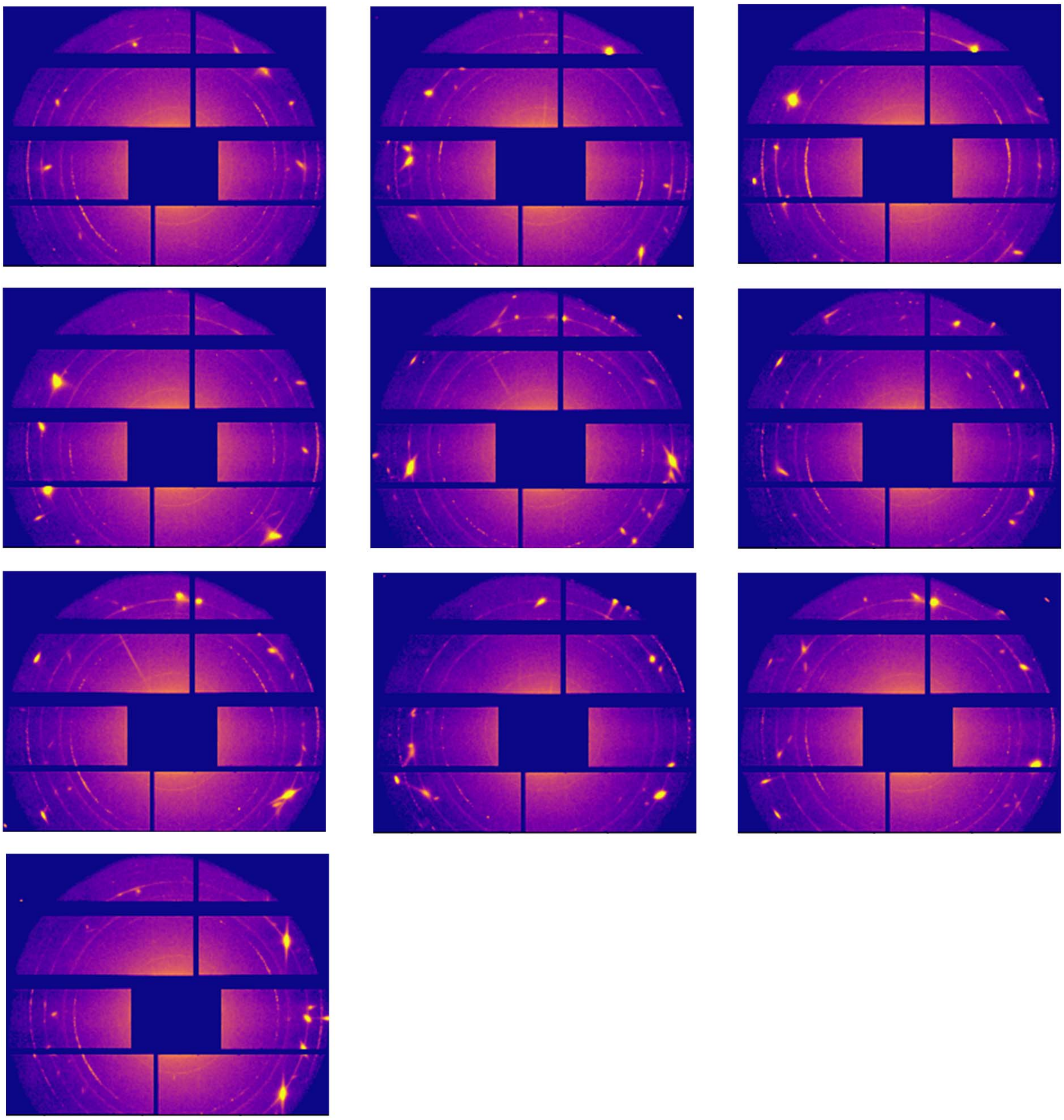

S2. SAXS and WAXS raw images for 10 different positions (0° rotation angle) for the 2618A alloy aged 2000 h at 473 K

S2.1. SAXS images

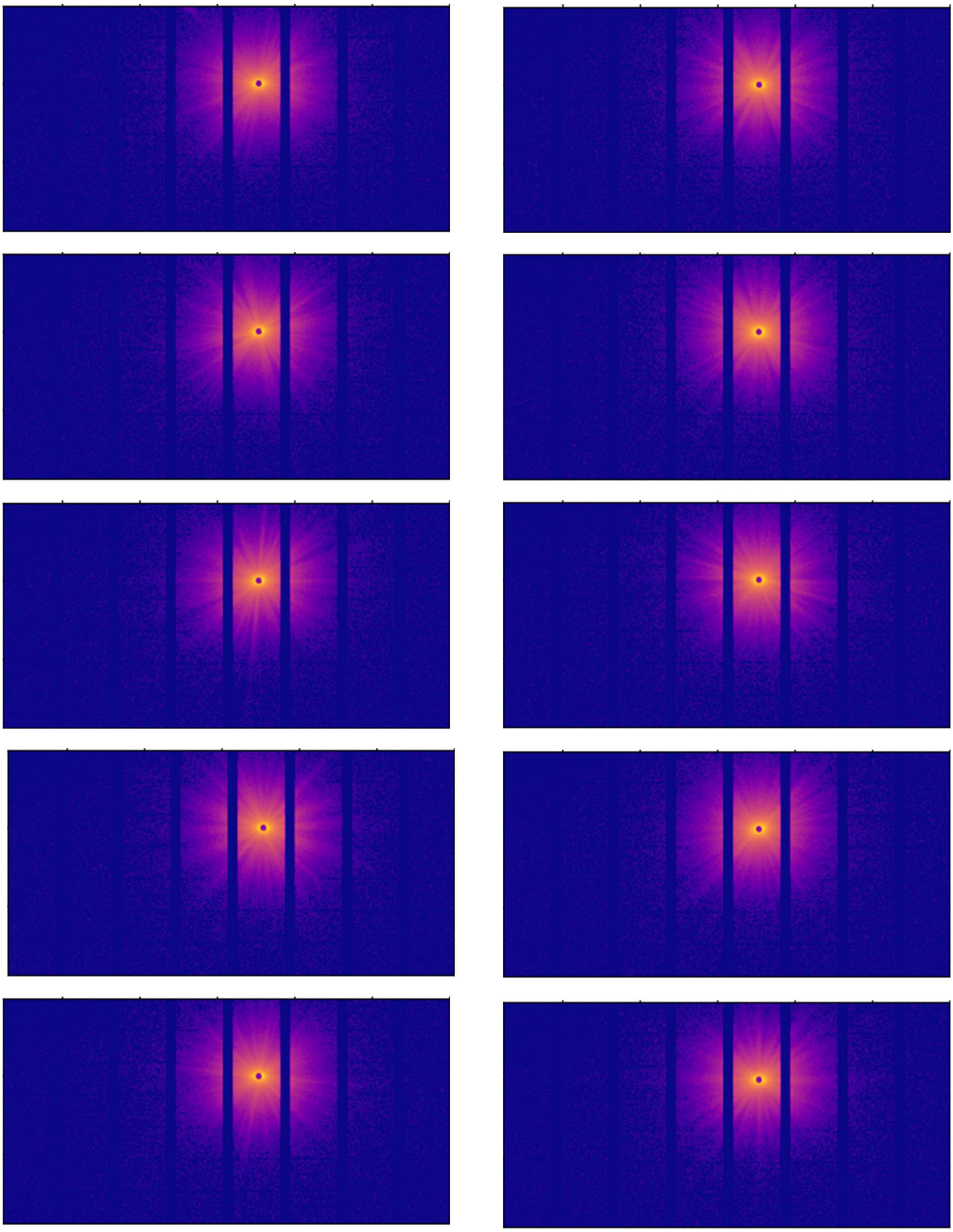

S2.2. WAXS images

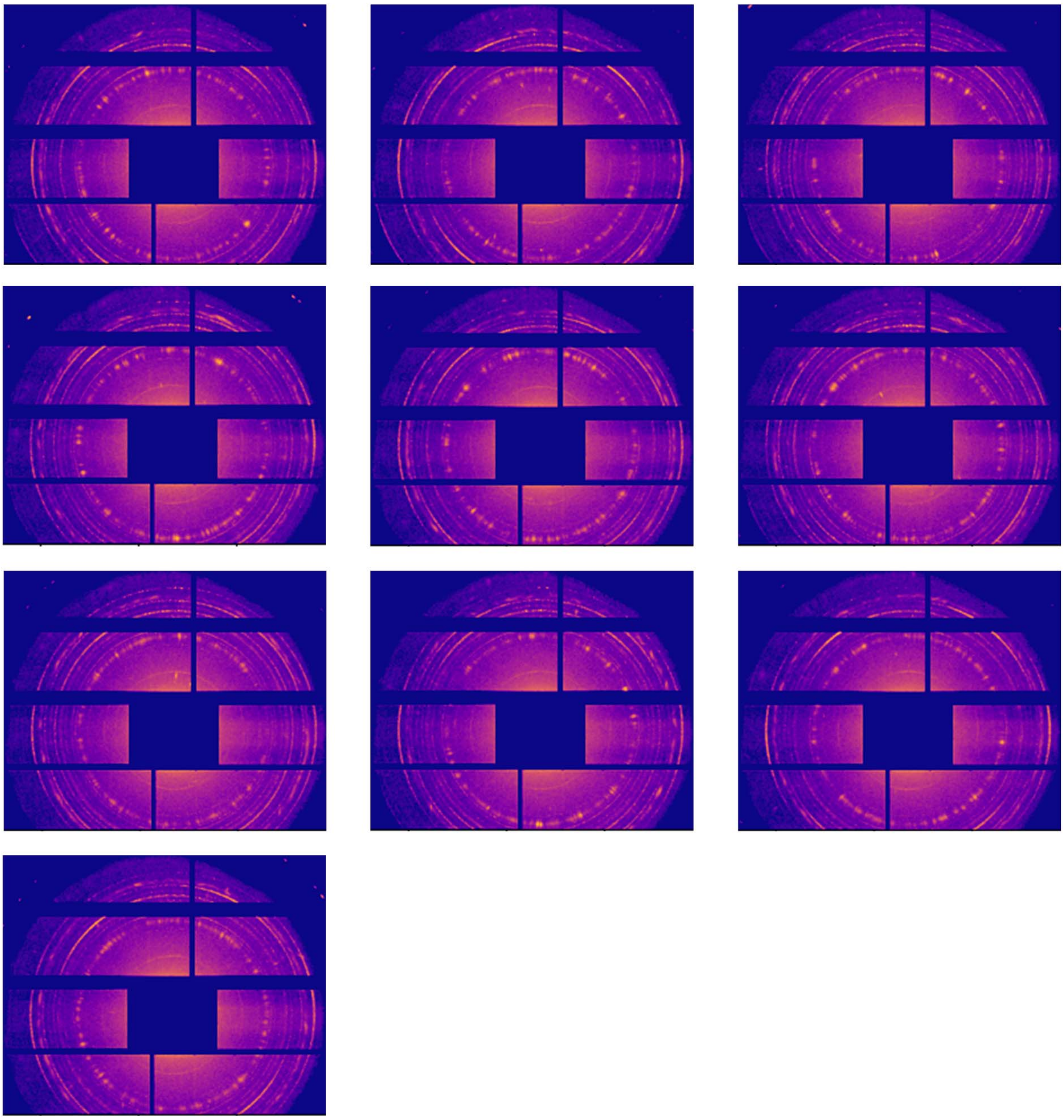

S3. Representation of the Ewald sphere for two orientations of a plate-like precipitate and a rod-like precipitate

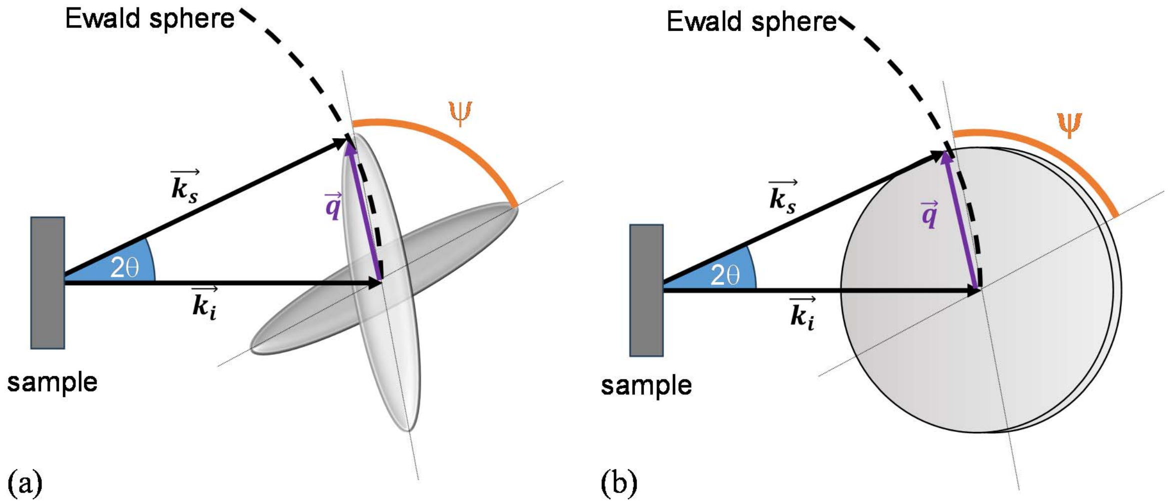

(a) Representation of the Ewald sphere for two plate-like precipitates with a tilt angle ψ between them. $\vec{k_i}$ is the incident wavevector, $\vec{k_s}$ the scattered wavevector, and $\vec{q}$ the scattering vector. (b) Representation of the Ewald sphere for two rod-like precipitates with the same nomenclature.

In the case of the plate-like precipitates, the orientation has a significant impact on the measured signal, with one orientation giving virtually no signal compared to the other, while for rod-like precipitates the signal remains unaffected by the tilt angle.

S4. Kinematic diagram and photograph of the experimental setup

Kinematic diagram with the four translation stages (Tr) and the rotation motor (Rot1) and photograph of the experimental setup showing the camera (3) used to position the sample rotation axis in front of the beam (1) and keep the distance between the sample (2) and the WAXS detector (4) constant.

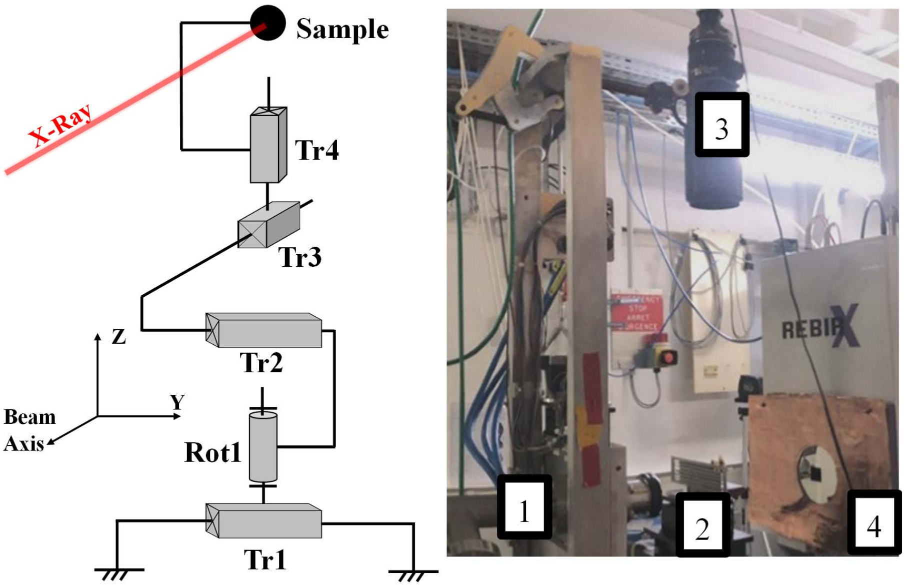

S5. SAXS and WAXS raw images for one position for each 3° angle and averaged along all angles for the 2219 alloy aged 2000 h at 473 K with the corresponding I(q) plots

S5.1. SAXS images

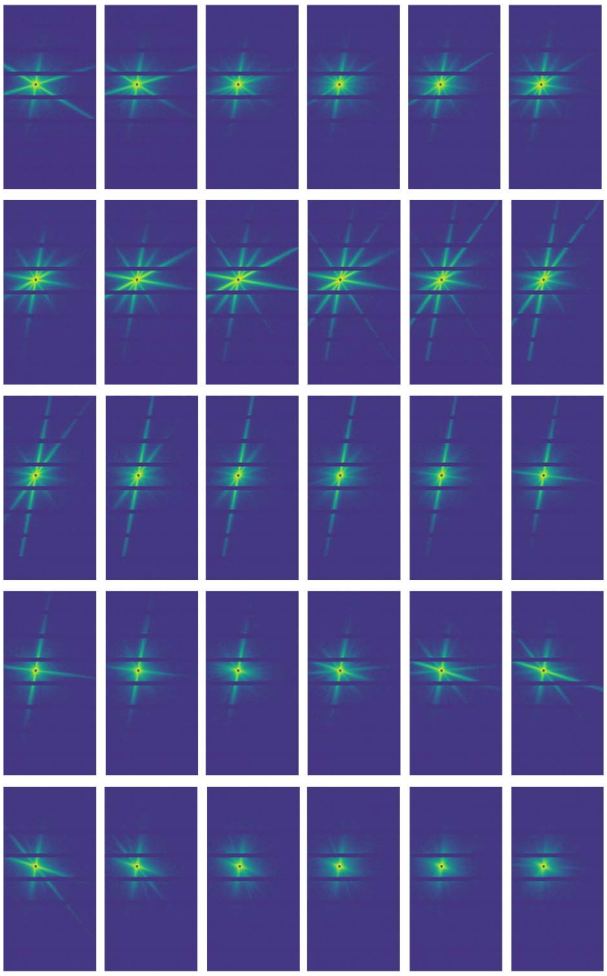

Raw image for each 3° angle

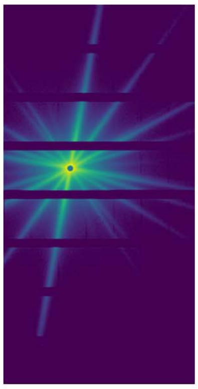

Image averaged along angles

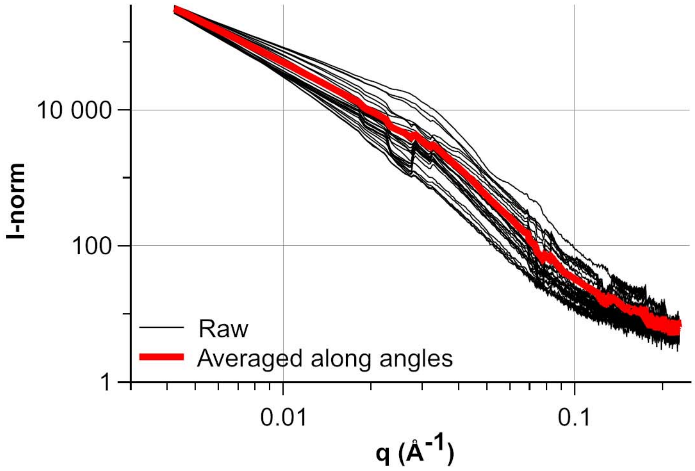

S5.2. WAXS images

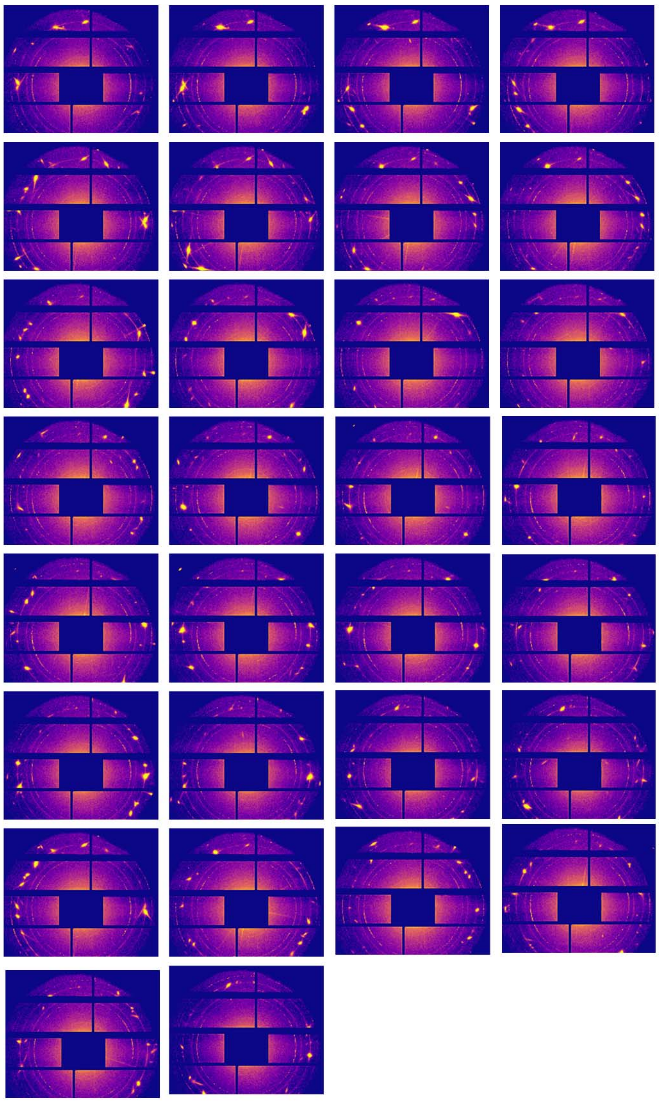

Raw image for each 3° angle

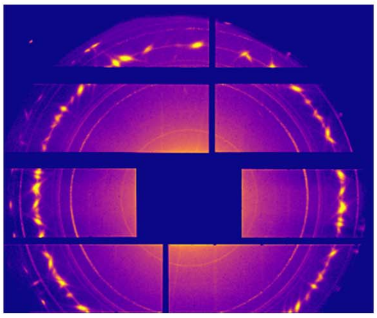

Image averaged along angles

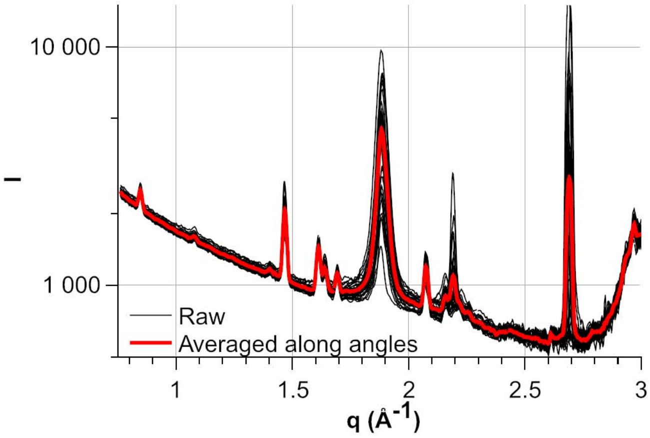

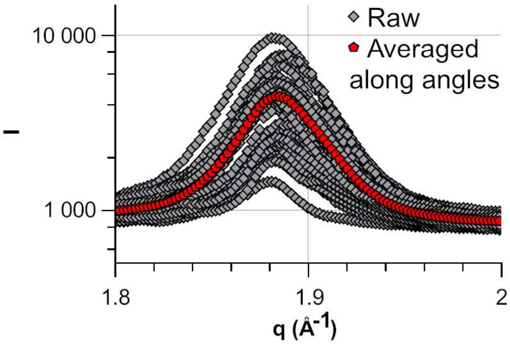

S6. SAXS and WAXS raw images for one position for each 3° angle and averaged along all angles for the 2618A alloy aged 2000 h at 473 K with the corresponding I(q) plots

S6.1. SAXS images

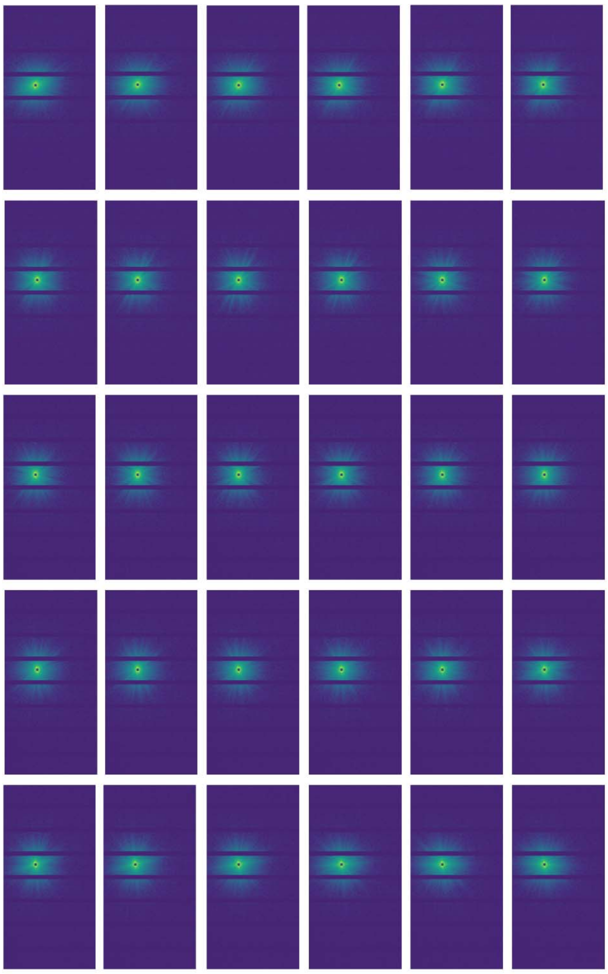

Raw image for each 3° angle

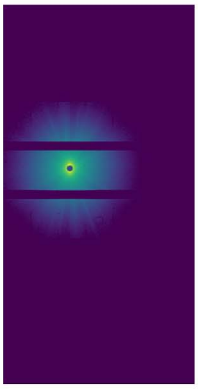

Image averaged along angles

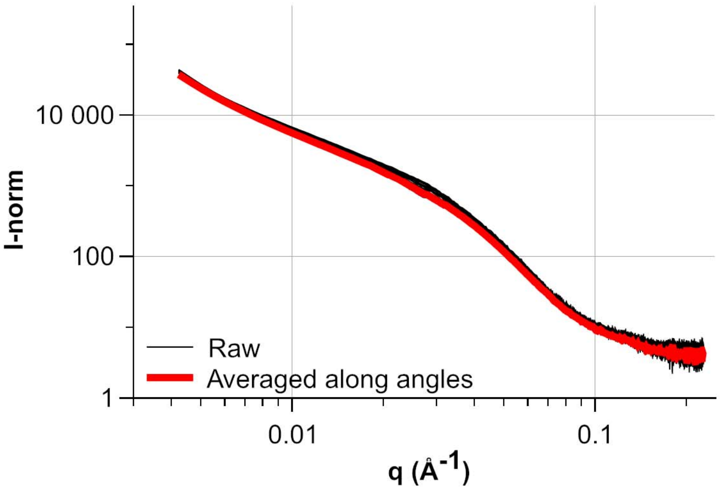

S6.2. WAXS images

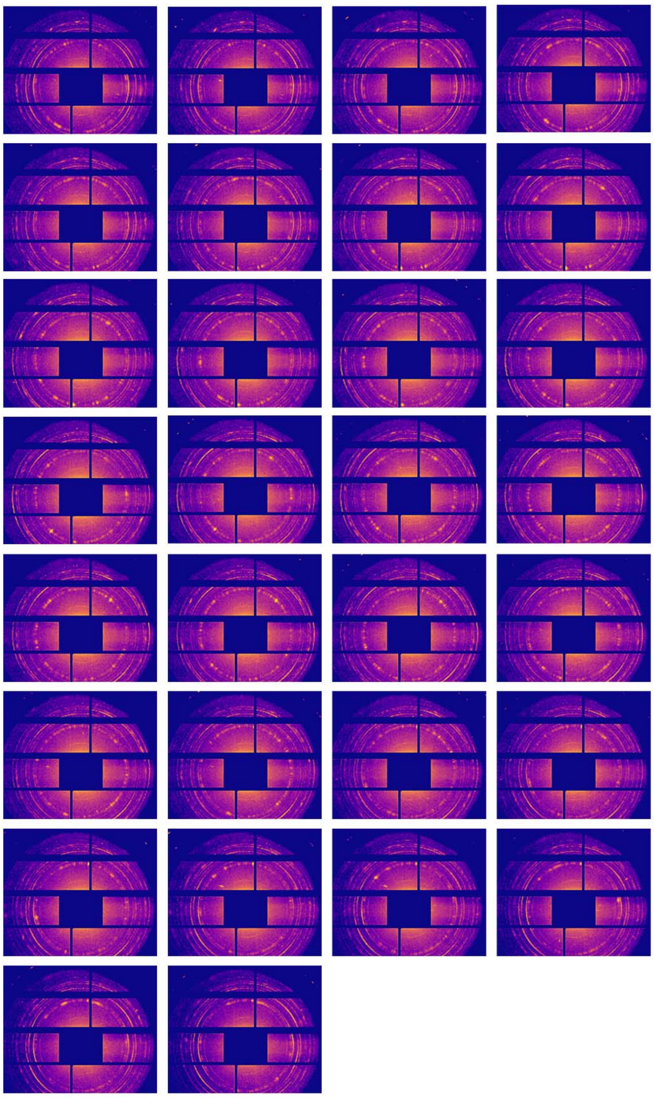

Raw image for each 3° angle

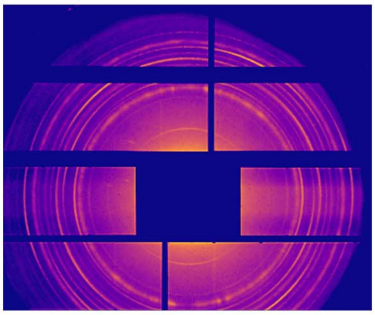

Image averaged along angles

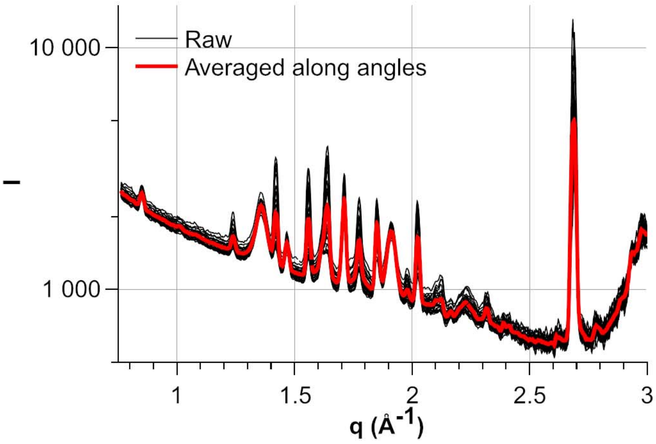

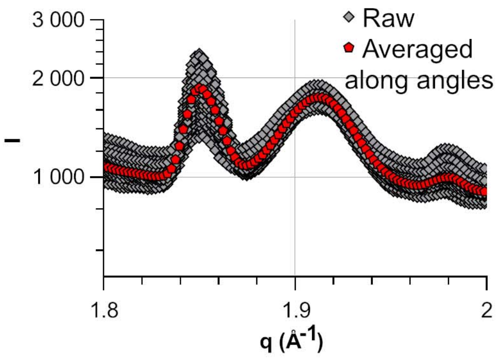

Supplement: Supplementary file 1 [file j-57-01800-sup1.pdf]
